# Fine-Tuning the Function of Farnesene Synthases for Selective Synthesis of Farnesene Stereoisomers

**DOI:** 10.1021/acs.jafc.4c09515

**Published:** 2024-11-26

**Authors:** Shengli Wang, Jiahui Zhou, Chuanling Zhan, Jianjun Qiao, Qinggele Caiyin, Meilan Huang

**Affiliations:** † Department of Pharmaceutical Engineering, School of Chemical Engineering and Technology, Tianjin University, Tianjin 300072, P. R. China; ‡ School of Chemistry & Chemical Engineering, Queen’s University Belfast, Northern Ireland BT9 5AG, U.K.; § Zhejiang Shaoxing Research Institute of Tianjin University, Shaoxing 312300, China

**Keywords:** isoprenoid, biosynthesis, function tuning, AlphaFold3, terpene synthase

## Abstract

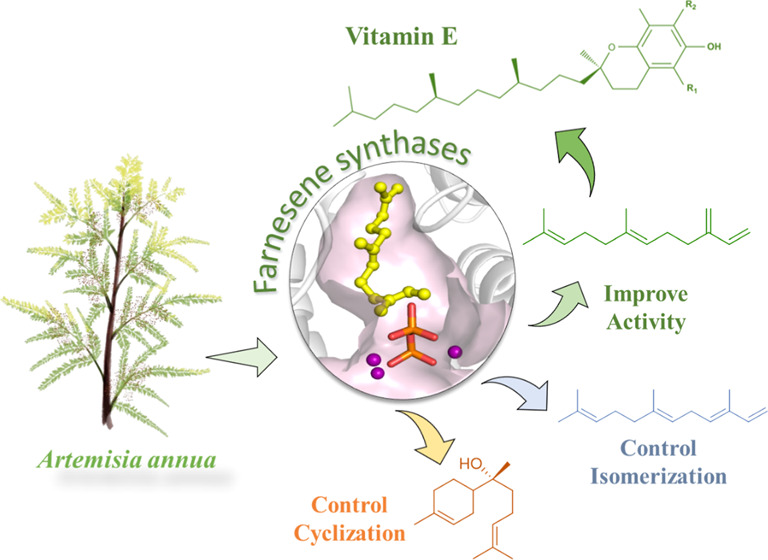

Farnesene synthase from *Artemisia annua* (AaFS) catalyzes the reaction from farnesyl pyrophosphate (FPP) to give the sesquiterpene β-farnesene, a key building block for the biosynthesis of vitamin E. However, an insufficient yield of β-farnesene precludes its industrialization. Understanding the mechanism would be essential for attaining β-farnesene in high yield. Guided by structure-based enzyme engineering, we designed several potent variants, among which L326I increased the β-farnesene yield from 450.65 to 3877.42 mg/L. Furthermore, we found that the function of β-farnesene synthase AaFS can be modulated at two positions; W299 is responsible for tuning the enzyme’s function to give its isomeric product α-farnesene and Y402 is the key residue for diverting from the linear farnesene products to the monocyclic α-bisabolol product. These findings provide valuable insights into the catalytic mechanism and functional modulation of farnesene synthases and set the basis for rational engineering of farnesene synthases for selective biosynthesis of diverse sesquiterpene natural products.

## 1 Introduction

Farnesene, first discovered in apple peels, is one of the simplest acyclic sesquiterpenes.^1^ It includes six stereoisomers belonging to α- and β-farnesene, respectively, among which 3 are naturally occurring isomers.^2^ Natural extraction of farnesene fails to meet the market demand because the content of farnesene in plants is extremely low.^3^ The farnesene obtained from chemical synthesis usually yields a mixture of isomers and byproducts.^4^ Overall, farnesene acquisition from plant extraction or chemical synthesis is associated with high cost, low production efficiency, and environmental pollution, which makes them unsuitable for industrial production. Therefore, microbial synthesis has become a promising alternative for farnesene production,^5−7^ and the best yield of β-farnesene has achieved up to 130 g/L in *Saccharomyces cerevisiae*.^6^

Farnesene is a valuable intermediate in the biosynthesis of various bioactive compounds, including vitamin E. Vitamin E is a powerful antioxidant and has been widely used in feed additives, medicine, food, cosmetics, etc. It contains eight fat-soluble natural isoforms, namely, α, β, γ, and δ isoforms of tocopherol and α, β, γ, and δ isoforms of tocotrienol.^8,9^ Among them, α-tocopherol is the most abundant and active isoform of vitamin E.^10^ Currently, more than 80% of commercial vitamin E is produced from chemical synthesis. Large-scale industrial synthesis of vitamin E (α-tocopherol) is traditionally fulfilled via two synthetic processes using either pseudoionone or linalool as the substrate.^11,12^ Recently, a new biofermentation process for synthesizing isophytol has been established using farnesene as the substrate.^5^ The structural difference of the farnesene isomers can influence the efficiency of their conversion to isophytol. The yield of farnesyl acetone (the intermediate of isophytol) was only 11% using α-farnesene as the substrate, while the yield was increased to 95% using β-farnesene.^5^ Compared to the traditional chemical synthesis process, the new process involves fewer reaction steps and uses fewer hazardous chemicals, reducing the carbon dioxide emissions by 60%.^2^ At present, 20% of the global vitamin E yield is produced using this new process. Thus, improving the yield of β-farnesene will make this process more competitive to be used for vitamin E production.

In microbial hosts, farnesene is produced from farnesyl pyrophosphate (FPP) via the heterologous expression of farnesene synthase (FS). FSs have been identified in various species, including *Malus domestica*,^13^*Artemisia annua*,^14^*Citrus junos,*^15^ and soybean^16^ and have been widely expressed in *Yarrowia lipolytica*,^17^*Escherichia coli*,^18^ or *S. cerevisiae*(5,6) for β-farnesene production, among which, AaFS from *A. annua* can catalyze the conversion of FPP to give a sole product (*E*)-β-farnesene.^14^ FSs are featured with three conserved motifs: aspartate-rich DDxxD motif, NSE/DTE motif, and H-1α loop motif.^19^ Site-directed mutagenesis analysis indicates that the DDxxD motif is essential for the catalytic activity of MdAFS of *M. domestica*.^20^ However, up to date, the crystal structure of FS has not been solved and only a few reports hypothesized the catalytic mechanism of FS.^20,21^ Thus, it is urgent to develop an efficient approach to identify the plastic residues in FSs and understand their effect on the enzymatic activity in order to rationally engineer the promising enzymes for efficient microbial production of β-farnesene.

Understanding the binding environments in favor of the formation of different farnesene products would enable us to reshape the binding pocket to achieve a high yield for β-farnesene. Here, we studied the binding of FPP in the *A. annua* β-farnesene synthase (AaFS), by protein modeling, molecular docking, and MD simulations. Enzyme engineering guided by the structural information showed that two variants M433I and L326I remarkably increased the catalytic efficiency of the enzyme. Furthermore, comparison of the structure of AaFS with the α-farnesene synthase from soybean (Fsso) disclosed that W299 is responsible for giving the α-farnesene stereoisomer, and Y402 is the key residue for forming the monocyclized α-bisabolol. These findings would set the structural basis for the modulating the farnesene biosynthetic pathways and rationally engineering FSs to give different farnesene stereoisomeric products, potentiating the industrial production of vitamin E from farnesene.

## 2 Materials and Methods

### 2.1 Strains and Media

*E. coli* DH5α (TransGen Biotech, Beijing, China) was used for routine plasmid construction and *E. coli* BL21 (DE3) (TransGen Biotech, Beijing, China) was used for recombinant protein expression. All *E. coli* strains were cultivated at 37 °C in Luria–Bertani (LB) medium (10 g/L tryptone, 5 g/L yeast extract, and 10 g/L NaCl) supplemented with 100 μg/mL ampicillin or 50 μg/mL kanamycin. Parent *S. cerevisiae* strain Sc027^27^ was used for in vivo characterization of FSs and was cultured at 30 °C in yeast extract peptone dextrose (YPD) medium (20 g/L glucose, 20 g/L tryptone, and 10 g/L yeast extract). Engineered yeast strains were cultivated in synthetic complete (SC) drop-out media (20 g/L glucose, 6.7 g/L yeast nitrogen base, 5 g/L (NH_4_)_2_SO_4_, and 2 g/L amino acid mix lacking uracil) at 30 °C.

### 2.2 Plasmids and Strains Construction

The encoding sequences of AaFS and Fsso (Table S1) were codon-optimized according to the *S. cerevisiae* codon bias and synthesized into yeast expression vector pESC-URA by GENEWIZ (Suzhou, China). For site-directed mutagenesis of FSs, an overlap-extension PCR was performed with pESC-AaFS or pESC-Fsso as a template. The amplified mutant fragments were ligated into the pESC-URA plasmid carrying the *GAL1* promoter and the *ADH1* terminator. DNA sequencing was performed to confirm that each mutagenesis occurred as expected. These plasmids for expression of FSs were individually transformed into *S. cerevisiae* Sc027 by the standard lithium acetate method.^28^ All of the plasmids and strains used in this study are listed in Tables S2 and S3, respectively. All of the primers used in this study are listed in Table S4.

### 2.3 Shake Flask Cultivation

The recombinant yeast strains were cultured in 10 mL of SC drop-out media at 220 rpm and 30 °C for 18 h. Subsequently, the culture solution was inoculated to 50 mL SC medium at an initial OD_600_ of 0.05 and grown at 30 °C and 200 rpm for 30 h. The galactose was then added with a final concentration of 10 g/L for the protein inducible expression, and 5 mL of dodecane was added to capture the product. After 120 h of cultivation, the dodecane phase was collected, filtered, and tested by gas chromatography–mass spectroscopy (GC–MS).^29^

### 2.4 GC–MS Analysis

The assay products were analyzed using the 8890–7000D GC–MS system equipped with a 7693A automatic liquid sampler (Agilent Technologies, California, USA). Gas chromatography was performed on an HP-5MS capillary column (30 m × 0.25 mm × 0.25 μm). One microliter of each dodecane sample was injected into the system with a split ratio of 1:10 and the carrier gas helium was set at a constant flow rate of 1 mL/min. The oven temperature was first maintained at 70 °C for 2 min and then gradually increased to 300 °C at a rate of 10 °C/min. The MS scan range (*m*/*z*) was 35 to 350.^30^ Standard compounds of β-farnesene (Sigma-Aldrich) were dissolved in dodecane and used to plot the standard curves for quantification.

### 2.5 Sequence Alignment

Multiple sequence alignments were performed using DNAMAN software, version 5.2.2 (Lynnon Biosoft, Canada). Sequence logos were created with the WebLogo (http://weblogo.berkeley.edu/).^26^

### 2.6 Heterologous Expression of AaFS and Fsso in *E. coli*

The encoding sequences of AaFS and Fsso (Table S1) were codon-optimized according to the *E. coli* codon bias and synthesized into the pET28a vector for protein expression by GENEWIZ (Suzhou, China). The pET28a–AaFS variant vector was obtained using overlap PCR method. The constructed plasmid was heterologously expressed in *E. coli* BL21 (DE3) and the transformant was grown in 10 mL of LB medium with 50 μg/mL kanamycin at 37 °C. Subsequently, 2 mL of seed culture was inoculated into fresh 200 mL of LB medium with 50 μg/mL kanamycin, followed by growth at 37 °C until the OD_600_ value reached 0.6. Following the addition of isopropyl β-D-1-thiogalactopyranoside (IPTG) to 0.2 mM to induce protein expression, the cultures were incubated for 20 h at 18 °C before being harvested by centrifugation (4000*g* for 40 min at 4 °C). The harvested pellet was resuspended in lysis buffer [20 mM Tris-HCl (pH = 8.0), 300 mM NaCl, 5 mM imidazole, and 10% glycerinum] and sonicated on ice for 30 min until the bacterial membrane was disrupted. The suspension was then centrifuged at 12,000*g* for 10 min at 4 °C. Proteins were then purified using NI-NTA Resin column (Genescript, Nanjing, China) according to the manufacturer’s instruction. The expected size of purified recombinant protein was confirmed by SDS-PAGE analysis.

### 2.7 Enzyme Kinetics

The kinetic parameters of wild-type AaFS (WT AaFS) and AaFS variants were determined according to published methods.^30^ The purified enzyme was incubated with FPP (Sigma-Aldrich) ranging from 2 to 80 μM into 100 mL of reaction buffer [50 mM Tris-HCl (pH = 8.0) and 10 μM MgCl_2_]. The reaction was allowed to proceed for 10 min at 30 °C and stopped by the addition of 100 μL solution (1 M EDTA and 4 M NaOH). Reaction products were extracted with 200 μL of hexanes and quantified based on the peak area ratio of assay products. Calculation of *K*_M_ and *k*_cat_ values was performed using nonlinear regression for the Michaelis–Menten model.

### 2.8 Modeling of Protein Structures

The apoprotein structures of AaFS and Fsso were generated using Modeler version 9.2^31^ (homology modeling), AlphaFold2,^32^ and AlphaFold3.^33^ The homology models were built based on the crystal structure of an α-bisabolol synthase (BOS) mutant (PDB ID: 5EAT,^34^ sequence identity: 42.28% and 31.33%, respectively). The locations of diphosphate (PPi) and three magnesium ions in the homology model and the AlphaFold2 model were taken from the crystal structure of (+)-bornyl diphosphate synthase from *Salvia officinalis* (PDB: 1N24(35)).

From the comparison, we found that the model for the WT AaFS built using AlphaFold3 looks more reasonable than homology modeling and AlphaFold2 (Figure S1). Specifically, the coordination of MG1 in the complex built by AlphaFold3 shows reasonable distances with the coordinating residues (≤2.4 Å), whereas the corresponding distances for MG1 in the complexes predicted by homology modeling or AF2 are slightly larger than typical coordinating distance. Therefore, we decided to use the structures generated by AlphaFold3 as our starting point for subsequent simulation.

The structures were further relaxed by 100 ns MD simulations. Then, the farnesyl cation was docked in the enzymes using AutoDock.^36^ Lamarckian genetic algorithm^37^ was used and 200 Lamarckian genetic algorithm runs were carried out for each enzyme complex. For each complex system, the top three representative poses were selected and subjected to another 100 ns MD simulations.

The systems were modeled using the ff19SB force field^38^ and general AMBER gaff force field^39^ for protein and substrates, respectively. The amino acid side chain protonation states were assigned using the H++ server.^40^ The protonation states of the key residues in the catalytic pocket were checked manually.^30,41,42^ The partial atomic charges and the missing force–field parameters of the substrate were obtained from the restrained electrostatic potential (RESP) charge using B3LYP/6-31G(d,p) implemented in the Gaussian16 package.^43^ The systems were solvated in a cuboid TIP3P water^44^ box with a minimum distance of 12 Å between any protein atom and the edge of the box. The AaFS system was neutralized by adding 24 Na^+^ counterions and the Fsso system was neutralized by adding 4 Na^+^ counterions.

5000 cycles of minimization (2500 cycles of steepest descent and 2500 cycles of conjugate gradient) were performed to relax the systems and then each system was gradually heated from 0 to 300 K over a period of 100 ps, followed by a 1 ns of *NVT* ensemble simulation at 300 K and 1 atm. In the heating and equilibration stage, positional restraints (force constant set to 5.0 kcal mol^–1^ Å^–2^) were applied to PPI, Mg^2+^ ions, and the coordinating residues. Subsequently, the constraints were released gradually in a further 100 ps simulation with the *NPT* ensemble (force constant decreases from 5.0 kcal mol^–1^ Å^–2^ to 0.0 kcal mol^–1^ Å^–2^ every 20 ps), and an additional 100 ns MD simulation with the *NPT* ensemble was performed to achieve the final equilibrium. The SHAKE algorithm^45^ was employed to constrain the bonds involving hydrogen atoms, and a time step of 2.0 fs was used for all simulations. The cluster analysis was conducted using Cpptraj tool^46^ from the last 10 ns equilibrated trajectories of all the 3 replicas of the MD simulations. Structures were collected by sampling at 100 ps intervals, and cluster analysis was performed based on the heavy atoms of the backbone to retrieve the representative snapshots of MD simulations. The most dominant cluster was used for the structure analysis. All molecular figures were created using PyMOL (The PyMOL Molecular Graphics System, Version 2.0. Schrodinger, LLC, 2015).

## 3 Results and Discussion

### 3.1 Catalytic Pocket of AaFS and the Substrate-Binding Mode

**Figure 1:**
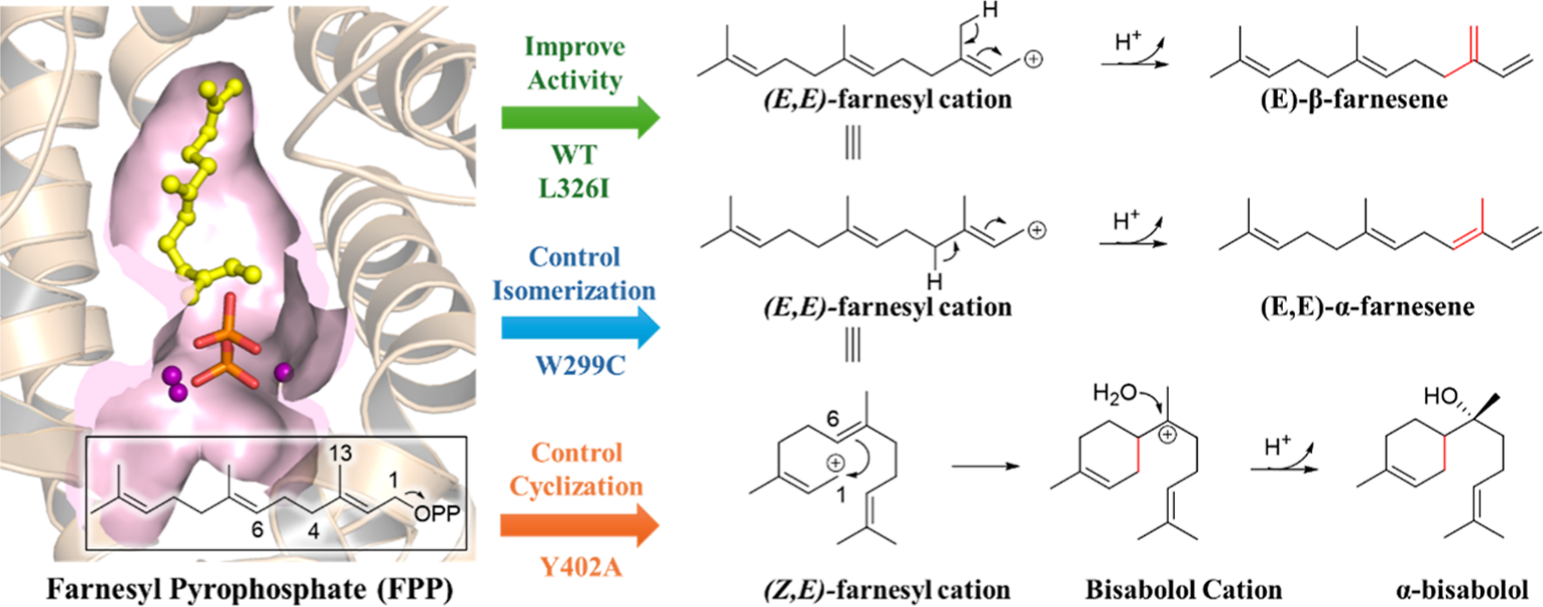
Fine-tuning the function of WT AaFS for FPP transformation to (1) improve the enzyme’s activity in yielding β-farnesene (green), (2) control the isomerization, yielding α-farnesene (blue), and (3) control the C1–C6 cyclization, giving α-bisabolol (orange).

AaFS gave the sesquiterpene β-farnesene, an important building block for the biosynthesis of vitamin E. However, the yield of plant β-farnesene biosynthesis is insufficient for industrial-scale fermentation. Further improvement of the yield of β-farnesene would potentiate the new biosynthesis process for vitamin E production from β-farnesene. On the other hand, different farnesene isomers could be achieved from the conversion of FPP via a common farnesyl cation intermediate. Understanding the mechanism for the formation of the representative sesquiterpene isomers would enable rerouting the function of AaFS for the synthesis of non-native bioproducts such as α-farnesene and the monocyclic bisabolol product (Figure 1).

**Scheme 1:**
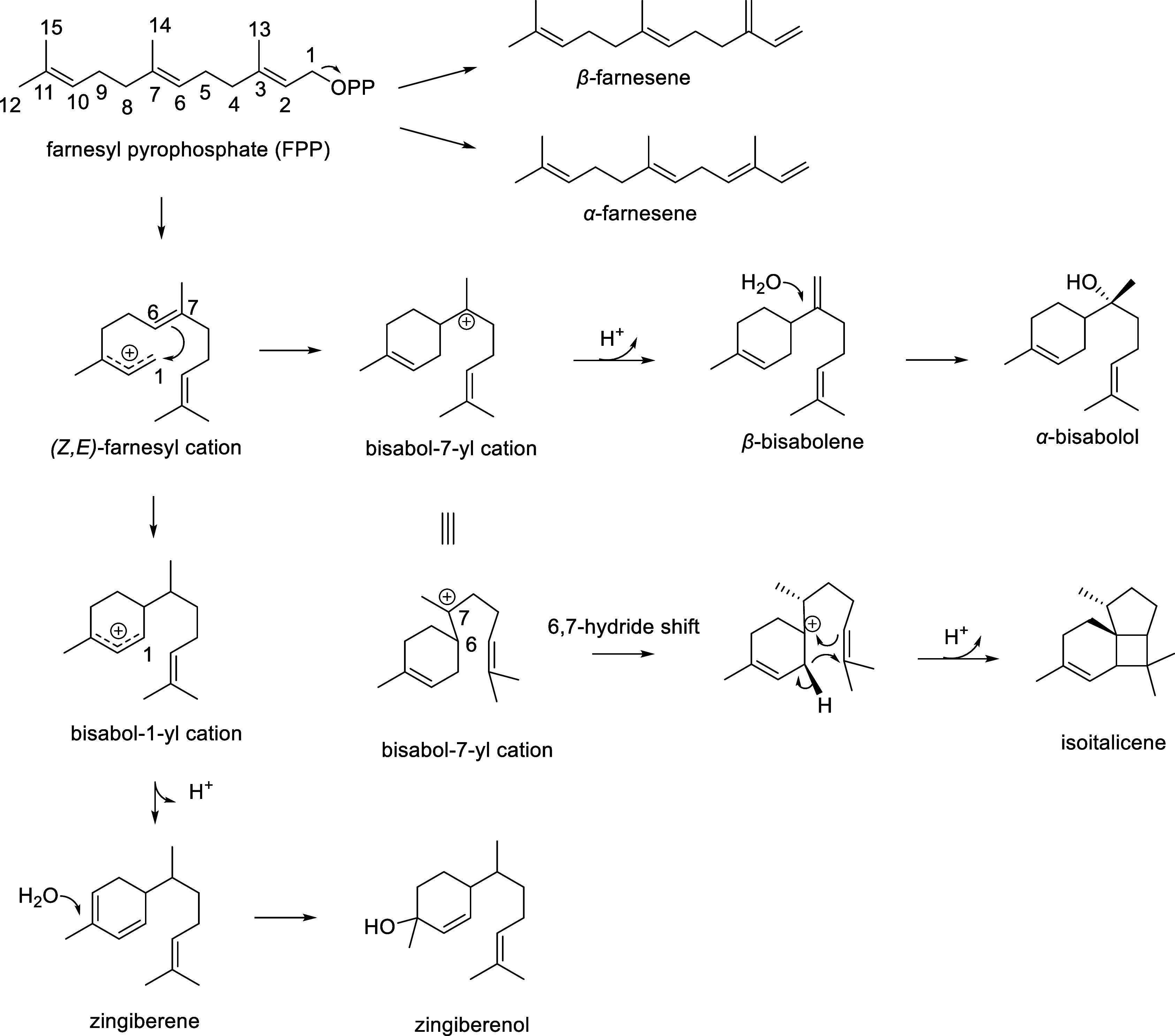
Proposed Reaction Pathways for the Products of WT AsFS and the Mutants Studied in This Research

The synthesis of farnesene starts with the loss of PPi from FPP to give the farnesyl cation, followed by the subsequent deprotonation. When catalyzed by α-farnesene synthase, FPP undergoes proton abstraction at the C4 position, resulting in the formation of the α-farnesene isomer; alternatively, when catalyzed by β-farnesene synthase, e.g., AaFS, the proton on C13 is abstracted by PPi, giving the β-farnesene product (Scheme 1).

When the proton on C4 is abstracted by PPi, it would give the α-farnesene isomer. The carbon cation around C1–C2–C3 allows the rotation of the C2–C3 single bond^22^ to give the (*Z*,*E*)-farnesyl cation. The farnesyl cation then undergoes 1,6-closure via electrophilic attack of the C6–C7 double bond by the positively charged C1 in the farnesyl cation, giving a bisabolyl cation intermediate, which is subjected to water molecule attack on the positively charged C7 to yield the α-bisabolol product.

**Figure 2:**
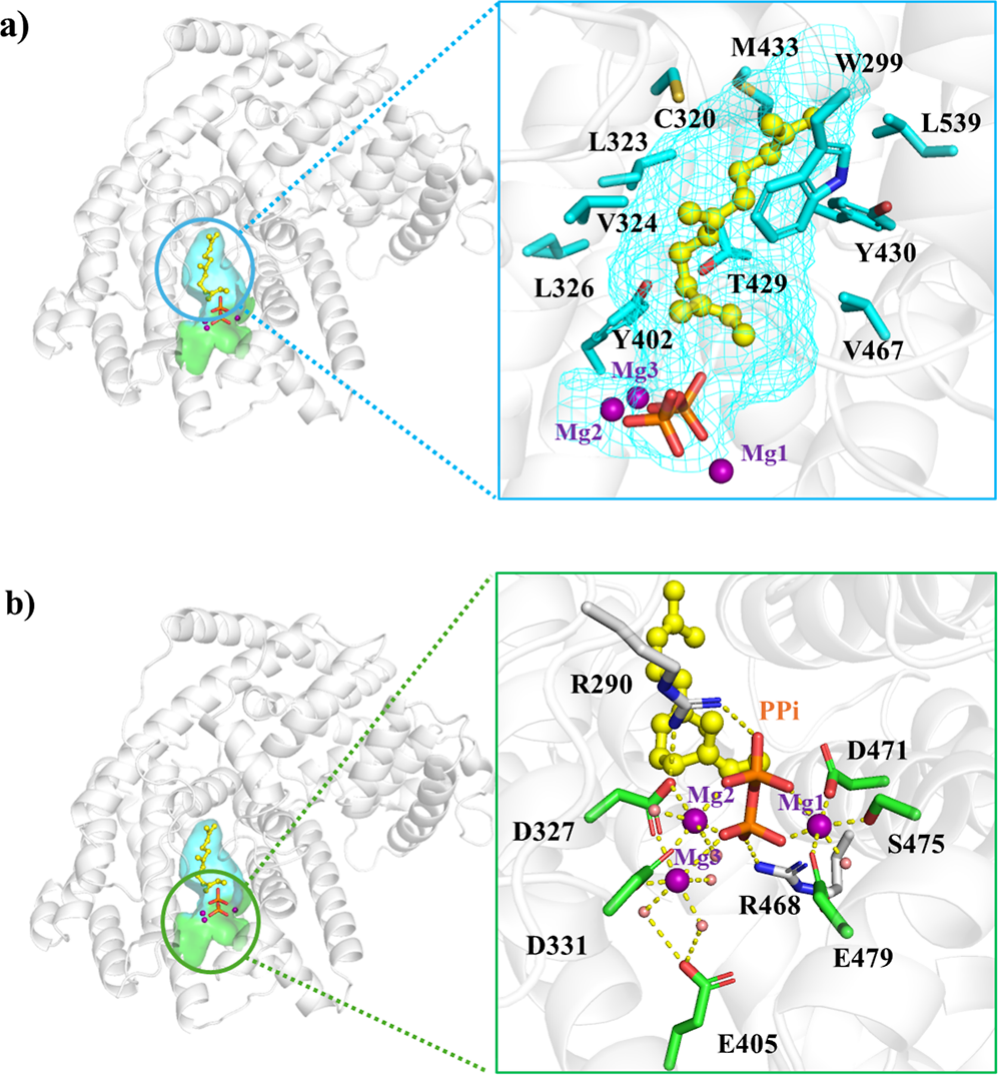
Binary catalytic site of WT AaFS. (a) Hydrophobic binding pocket around the substrate. (b) Hydrophilic region around the magnesium ions and PPi.

Magnesium ions usually play an important role in stabilizing the binding of phosphate groups and facilitating the formation and cyclization of key intermediate carbon cations.^23,24^ From MD simulations, we disclosed the binding environment of the three magnesium ions and farnesyl cations in AaFS (Figure 2). Mg1 is coordinated with the DSE motif comprising D471, S475, E479, both phosphate of PPi, and a water molecule. Mg2 and Mg3 are coordinated with the conserved aspartic acids D327 and D331 in the DDxxD motif. Mg2 is coordinated with two oxygen atoms of the α- and β-phosphate oxygen atoms of PPi; Mg3 is coordinated by β-phosphate oxygen of PPi and its remaining coordination is coordinated by 3 water molecules, two of which form H-bonds with E405. The α- and β-phosphates of PPi are stabilized by R468 and R290, respectively. R290 interacts with Mg-coordinating D327. The farnesene chain is stretched out and bound within a hydrophobic pocket composed of W299, C320, L323, V324, L326, Y402, T429, Y430, M433, V467, and L539 (Figure 2).

AaFS belongs to Type I terpene synthases in which Mg1 is usually coordinated with an NSE/DTE motif.^23,25^ Interestingly in WT AaFS, Mg1 is coordinated with a DSE motif (Figure 2b), which is located in the same region as the typical NSE/DTE motif in sesquiterpene synthases. To examine if AaFS can accommodate NSE or DTE motifs instead of the DSE motif adopted in the wild type, D471 was mutated to N471 and S475 was mutated to T475. We found that the yield of β-farnesene was significantly reduced in the D471N variant, while it was maintained in the S475T variant (Table S5), indicating that AaFS prefers the DSE and DTE motifs for Mg1 coordination.

To verify the binding environment of the magnesium ion and PPi, E405 and R468 were mutated to alanine. MD simulated structures of the enzyme variants E405A and R468A display unusual penta-coordination (Figure S2a,b). The E405A and R468A mutations disrupted the favorable electrostatic interactions that stabilize Mg-coordinating water and PPi, therefore resulting in unusual penta-coordination. The unstable penta-coordination accounts for the reduced enzyme activities as observed in the experiment (Figure S2c).

### 3.2 Engineering AaFS to Improve the Yield of β-Farnesene

**Figure 3:**
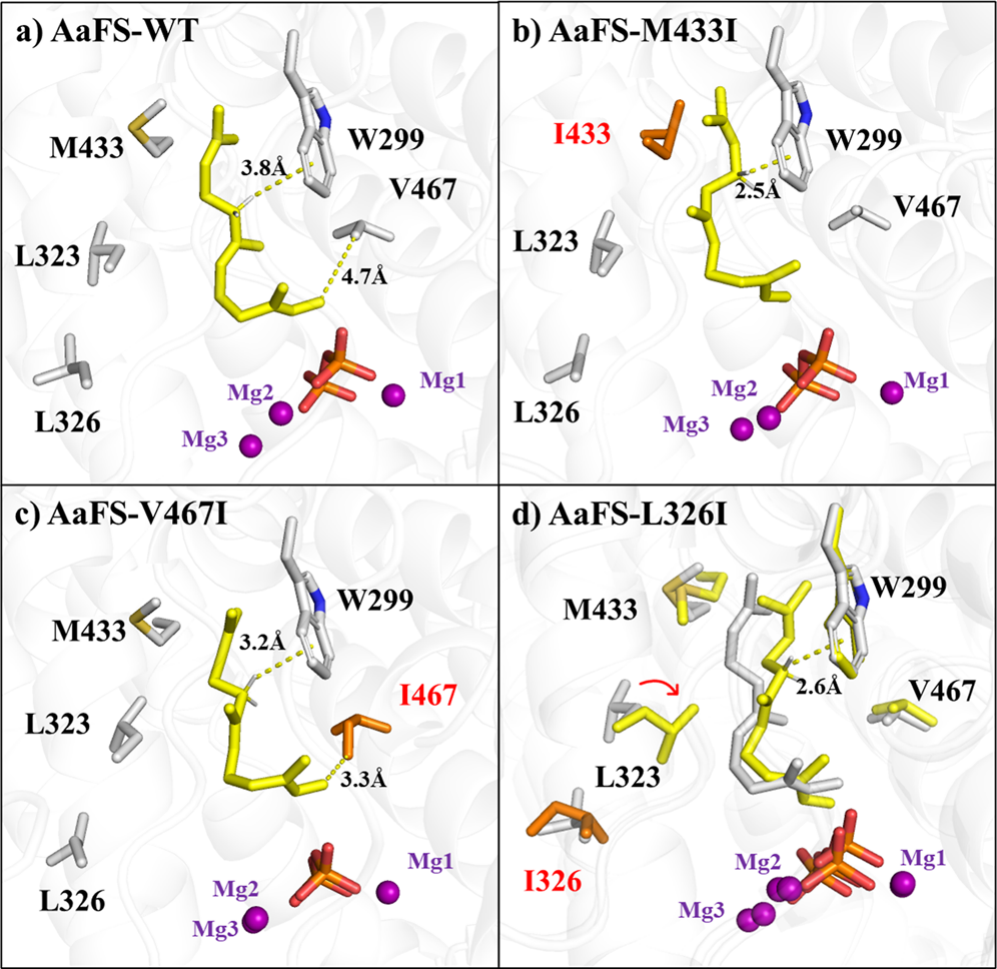
Reshaping the hydrophobic pocket around the substrate in the AaFS. (a) WT AaFS; (b) AaFS-M433I; (c) AaFS-V467I; and (d) AaFS-L326I. The structures of AaFS-L326I (yellow) and WT AaFS (gray) are superimposed to demonstrate the different orientations of L313 critical for reshaping the pocket.

In order to improve the yield of β-farnesene, based on the substrate-binding mode in the WT AaFS (Figure 2b) and multiple sequence alignment of 433 homologous sequences (Figure S3), we reshaped the binding pocket by mutating residues located at the active site. Through experimental verification, the L326I, M433I, and V467I variants showed increased enzyme activity, resulting in β-farnesene yields of 3877.42, 2961.13, and 767.65 mg/L (Table 1, Figure S4), respectively, representing 8.6-fold, 6.6-fold, and 1.7-fold increases compared to the WT AaFS. MD simulations demonstrated that in the WT enzyme, W299 forms a stable hydrophobic interaction with the substrate molecule (Figure 3a). This interaction restricts the aliphatic chain of the substrate from folding, thereby reducing the competitive formation of cyclized products and improving the binding affinities. The mutations in the M433I and V467I variants significantly enhanced the hydrophobic interaction between W299 and the substrate (Figure 3b,c). In the enzyme variant L326I, the side chain of L323 flipped, forming a hydrophobic interaction with the substrate, which in turn interacts with W299 via a stable hydrophobic interaction (Figure 3d).

**Table 1:** Steady-State Kinetic Parameters of AaFS

enzyme	*K*_M_ (μM)	*k*_cat_ (s^–1^)	*k*cat/*K*M (μM^–1^·s^–1^)
WT AaFS	14.34 ± 2.3	1.74 ± 0.12(× 10^–1^)	12.1 × 10^–3^
AaFS M433I	7.64 ± 4.8	2.49 ± 0.17 (× 10^–1^)	32.6 × 10^–3^
AaFS L326I	6.75 ± 3.7	2.72 ± 0.12 (× 10^–1^)	40.4 × 10^–3^
AaFS W299C	21.29 ± 1.8	0.96 ± 0.22 (× 10^–1^)	4.51 × 10^–3^

Steady-state kinetic analysis was conducted for the variants L326I and M433I, which showed the most enhanced activities compared to the WT enzyme, and also for W299C, which modulated the function of AaFS to become an Fsso-like terpene synthase (Table 1). The catalytic efficiency of the best-performing variant L326I is 3.3-fold higher than that of the WT. Conversely, disrupting the CH-pi interaction between W299 and the substrate significantly reduces the catalytic efficiency.

Exploiting statistical amino acid frequencies from multiple sequence alignments has been a useful method to guide for engineering the catalytic activity of enzymes.^26^ Except for the aforementioned residues that surround the binding sites, we also considered some distal sites, CbD (conserved but different) sites that are conserved in 37 FS protein sequences but different in the AaFS protein sequence (Figure S5 and Table S6). Then, 15 CbD sites of AaFS were selected and mutated to the equivalent consensus residues for subsequent rational engineering, among which Q142H displayed a 3-fold increase in β-farnesene yield (Figure S6).

### 3.3 Turning the Catalytic Site of AaFS for the Isomerized Product

**Figure 4:**
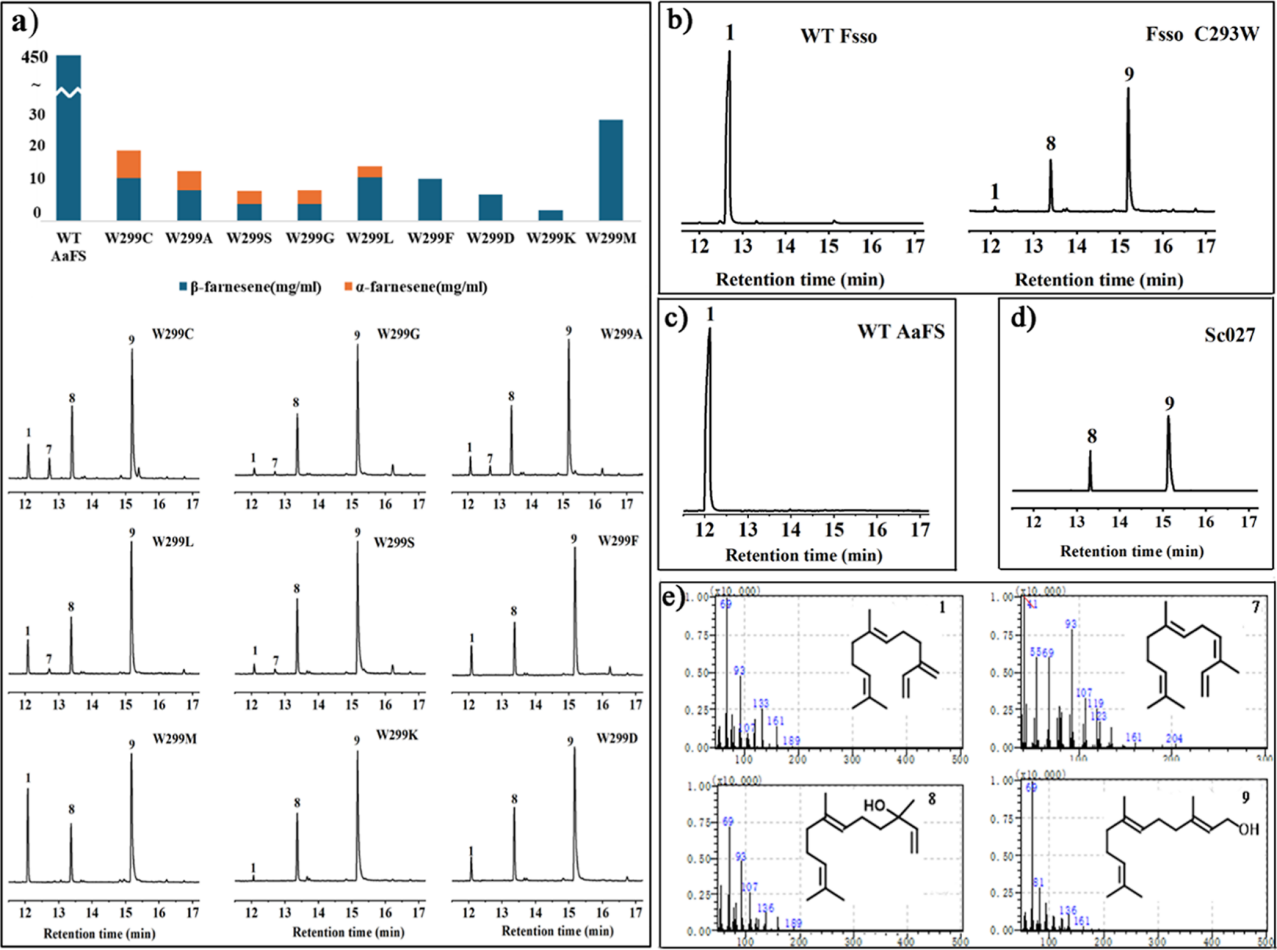
Product characterization by GC–MS. (a) β-farnesene yield by AaFS W299 variants; (b) WT Fsso and Fsso C293W variants; (c) WT AaFS; and (d) Sc027 strain. Sc027 has been engineered by overexpressing MVA pathway-related genes in parent CEN.PK2.1C. So, the downstream products of FPP in the MVA pathway, nerolidol and farnesol, were detected in Sc027 strains without plasmids; (e) MS spectra corresponding to the sesquiterpene peaks. Produced sesquiterpenes were assigned as (1) β-farnesene; (7) α-farnesene; (8) nerolidol; and (9) farnesol.

To validate the effect of W299 that stabilizes the aliphatic chain of the substrate, W299 was mutated into less bulky residues (Cys, Ala, Ser, Gly, Leu, Phe, Asp, Lys, and Met), and they showed reduced yield of β-farnesene (Figure 4a). Interestingly, α-farnesene was observed along with β-farnesene, when W299 was mutated into smaller residues Cys, Ala, Gly, Ser, and Leu (Figure 4a). Noting that α-farnesene is the sole product of Fsso, we hypothesized that mutating this key residue at W299 would tailor the function of AaFS to make it similar to Fsso.

Fsso and AaFS share a sequence identity of 30.40%. Fsso catalyzes the synthase of α-farnesene also via the farnesyl cation intermediate.^16^ During the catalysis, the proton on C4 is abstracted by PPi to give the α-farnesene product (Figure 1).

**Figure 5:**
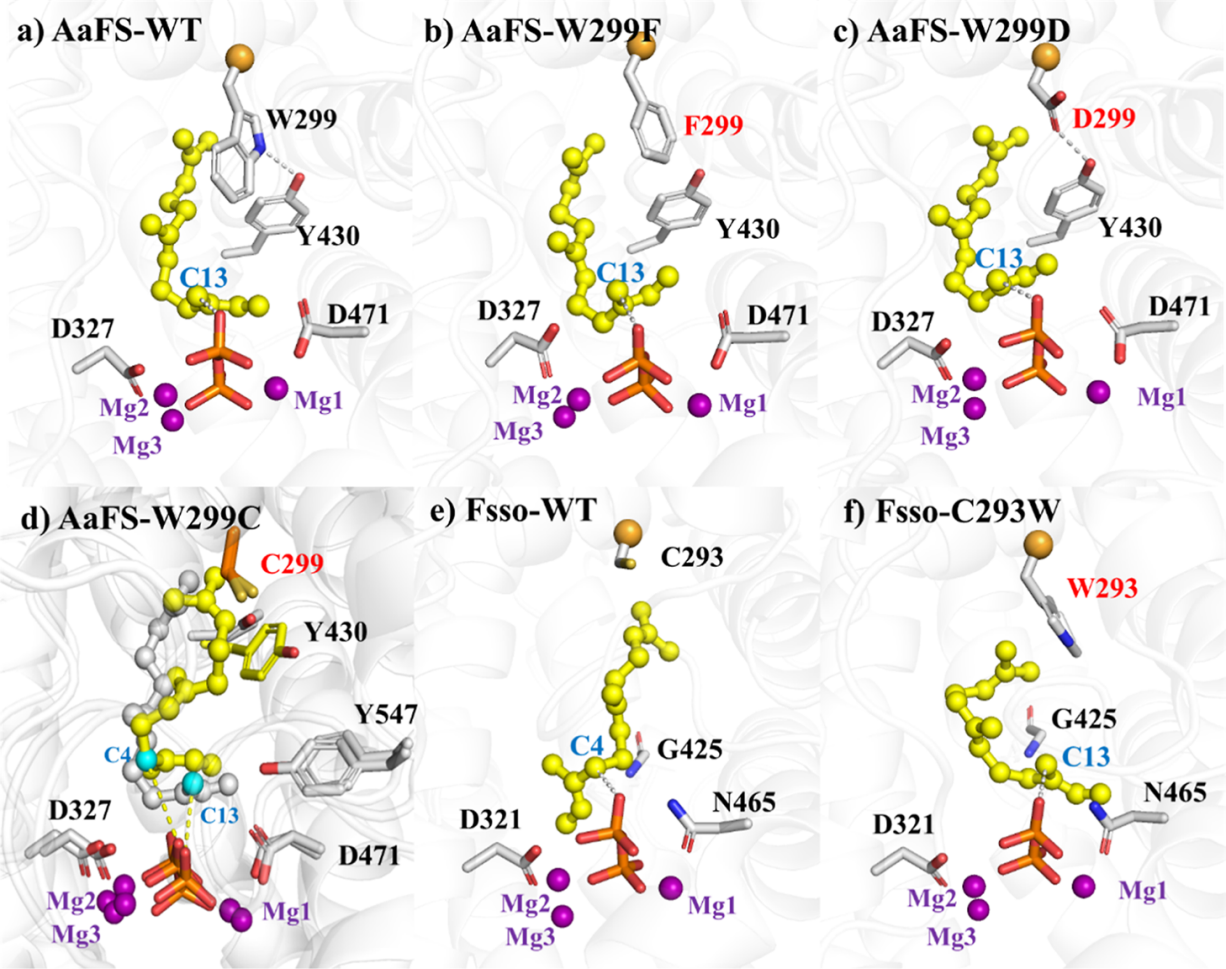
Engineered AaFS and Fsso with altered functions in the production of farnesene stereoisomers. (a) AaFS-WT, (b) AaFS-W299F, (c) AaFS-W299D, and (d) AaFS-W299C. Two conformations of farnesyl cation are observed; one liable to give β-farnesene (gray) with C13 closer to the PPi and the other conformation liable to give α-farnesene (yellow) with the C4 atom closer to the PPi. The C13 atom in the pro-β-farnesene conformation and the C4 atom in the pro-α-farnesene conformation are shown in blue. (e) Fsso-WT and (f) Fsso-C293W.

By comparing the simulation results of β-farnesene synthase WT AaFS and α-farnesene synthase WT Fsso, we observed that the steric hindrance of W299 and Y430 in AaFS may force the substrate to adopt a curved “C”-type conformation (Figure 5a). This conformation allows the C13 atom of the substrate to approach PPi for deprotonation, resulting in the formation of β-farnesene (1) (see the product spectrum in Figure 4c). In contrast, in Fsso, the corresponding residue at position 299 is a smaller cysteine, and the reduced steric hindrance compared to tryptophan allows the substrate to adopt an extended “E”-type conformation (Figure 5e). In this conformation, the C4 atom of the substrate is positioned in proximity to PPi, favoring deprotonation of C4 to and the formation of α-farnesene, the sole product (7) in WT Fsso observed in experiments (Figure 4b).

Mutating W299 into a less bulky residue, such as Phe, does not completely eliminate steric hindrance; the substrate still adopts the “C”-type conformation as in WT-AaFS (Figure 5b). Mutating W299 to Asp resulted in a hydrogen bond with Y430, which also maintains steric hindrance (Figure 5c).

The corresponding residue at W299 in the α-farnesene synthase Fsso is cysteine; therefore, we mutated W299 to cysteine in order to modulate the farnesene product to give α-farnesene. Our MD simulation disclosed that replacing tryptophan at position 299 with cysteine reduced steric hindrance, allowing the aliphatic chain of farnesyl cation to adopt different conformations: one liable to give β-farnesene (Figure 5d gray) and the other liable to give α-farnesene (Figure 5d yellow). As expected, experiments showed that the production of both α-farnesene (6.68 mg/L) and β-farnesene (10.55 mg/L) were achieved (Figure S7).

In contrast, when C293 in Fsso was mutated into the corresponding tryptophan in AaFS, the bulky tryptophan residue hinders the terminus of the substrate aliphatic chain from approaching the bulky residue to form the extended conformation (Figure S8) and only the pro-β-farnesene conformation can be accommodated (Figure 5f), so that exclusively β-farnesene product was observed (Figure 4b).

### 3.4 Turning the Catalytic Site of AaFS for Producing α-Bisabolol

**Figure 6:**
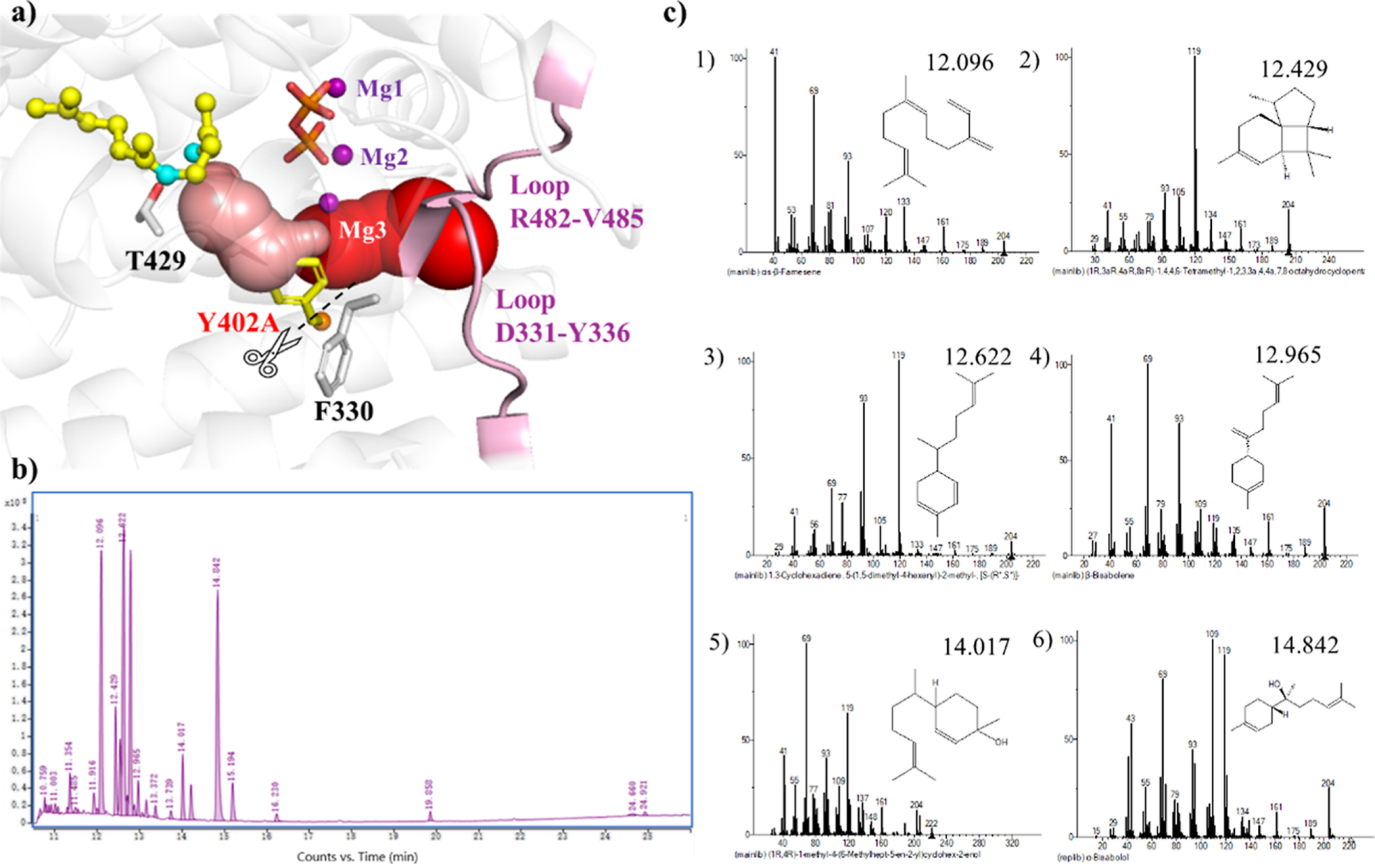
Product characterization of the AaFS-Y402A variant. (a) MD simulated structure of Y402A. The water channel is represented by spheres and the extended portion shown in pink represents the water around the substrate. The position of Y402 in the WT AaFS is illustrated by superimposition of the variant and WT AaFS structures. (b) MS spectra corresponding to the sesquiterpene peaks of the AaFS-Y402A variant. (c) Produced sesquiterpenes were identified as (1) β-farnesene; (2) isoitalicene; (3) zingiberene; (4) β-bisabolene; (5) zingiberenol; and (6) α-bisabolol. The reaction pathways of the products are illustrated in Scheme 1.

MD simulations of AaFS disclosed the binding mode of the substrate (Figure 2b). Y402 pointed toward the C5–C6 bond of the substrate, restricting the folding of the aliphatic chain to give a monocyclic product by 1–6 closure (Figure S9a). Mutating the bulky Y402 to small alanine would give extra space, allowing the folding of the aliphatic chain to give a monocyclic product. To reduce the steric hindrance at position 402 in AaFS, Y402 was replaced with alanine. MD simulations of the enzyme variant Y402A showed a folded structure, where C1 is in proximity to C6, favorable for the subsequent 1,6 closure (Figure S9b). Additionally, the extra space created by the Y402A mutation allows the entry of water molecules in the proximity of the substrate, forming a water channel passing through a gate between two exposed loops ^331^DNYGTY^336^ and ^482^RGHV^485^ (Figure 6a). With the time evolution of MD simulations, additional water molecules enter the catalytic site and retain around the substrate via the H-bond interaction with T429, forming a water channel (Supporting Information video). We also added a figure (Figure S9e) to show the presence of constant hydrogen bonds between T429 and the water.

We evaluated the Y402A variant by experiments. As expected, in addition to the β-farnesene product, monocyclic products (Figure 6c) were also observed, including two hydroxylation products zingiberenol and α-bisabolol, because of the presence of the aforementioned water channel. It is worth noting that a new tricarbocyclic product isoitalicene (2) was observed, which may be obtained by further cyclization of the bisabolyl cation intermediate preceded by 6,7-hydride shift (Scheme 1).

In addition, we further mutated the residues interacting with Y402, such as F330, L398, and T427 (Table S5), and found that F330A allowed the flexibility of Y402, also creating extra space (Figure S9c) and hence giving the new isoitalicene product (Figure S10).

The bottleneck of the industrial-scale production of vitamin E from β-farnesene is to improve the yield of sesquiterpene β-farnesene. Understanding the mechanism of generating divergent isomeric farnesene products would enable us to reshape and optimize the binding environment of FPP to enhance the β-farnesene yield.

However, the mechanism of the enzyme is elusive because the structure of the FS is not available. By combining molecular simulations and validated by mutagenesis studies, we unveiled the binding environment of the farnesyl cation in the presence of PPi and three magnesium ions in β-farnesene synthase AaFS. Guided by the substrate-binding mode in the WT enzyme, we engineered the β-farnesene synthase AaFS and achieved several potent variants with a remarkably increased yield of β-farnesene, including L326I and M433I in the catalytic site.

The structural comparison between AaFS and the α-farnesene synthase Fsso indicated that W299 and Y402 are crucial for their function to give different farnesene isomers; mutating W299 in β-farnesene synthase AaFS into cysteine gives the isomeric α-farnesene product, while mutating C299 in α-farnesene synthase Fsso into tryptophan gives non-native β-farnesene. On the other hand, mutating Y402 of AaFA into smaller residues like alanine caused the substrate to adopt a folded conformation and allowed the entrance of the water, diverting the biosynthetic routes from the farnesene products, and resulting in the production of the monocyclic α-bisabolol isomeric product.

The research reported here sets the structural basis for the rational engineering of FSs for the biosynthesis of valuable isoprenoid natural products.

## Supporting Information Available

Coding sequences of yeast codon-optimized genes; overview of plasmids used in this study; strains used in this study; primers used in this study; yields of β-farnesene of AaFS variants; protein sequences of FSs used for WebLogo analysis; comparison of models from three modeling methods; binding environment of FPP in AaFS variants; WebLogo plot based on multiple sequence alignment; electropherogram of the SDS-PAGE; propensity in amino acid residues in the sequences of AaFS and its 37 orthologues; site-directed mutagenesis of AaFS; product identification and farnesene production of AaFS W299C variant; conformational difference of the farnesyl cation substrate between Fsso-WT and Fsso-C293W; substrate binding poses in AaFS WT and variants; and AaFS-F330A MS spectra (PDF)
Water channel in AaFS-Y402A (MP4)
