# Supplementary material for: Fine-Tuning the Function of Farnesene Synthases for Selective Synthesis of Farnesene Stereoisomers
Source: J Agric Food Chem. 2024 Nov 26;72(49):27355–64. doi: 10.1021/acs.jafc.4c09515 (PMC11638952; doi:10.1021/acs.jafc.4c09515)
Supplement: Supplementary file 1 — jf4c09515_si_001.pdf [file jf4c09515_si_001.pdf]

**Supplementary information**

# Fine-Tuning the Function of Farnesene Synthases for Selective Synthesis of Farnesene Stereoisomers

Shengli Wang[1,3#], Jiahui Zhou[2#], Chuanling Zhan[1,3], Jianjun Qiao[1,3*], Qinggele Caiyin[1,3*], Meilan Huang[2*]

[1]Department of Pharmaceutical Engineering, School of Chemical Engineering and Technology, Tianjin University, Tianjin 300072, P. R. China

[2]School of Chemistry & Chemical Engineering, Queen's University Belfast, BT9 5AG, Northern Ireland, United Kingdom

[3]Zhejiang Shaoxing Research Institute of Tianjin University, Shaoxing 312300, China

[#] Shengli Wang and Jiahui Zhou contributed equally to this work

[*] Corresponding author: Meilan Huang; Qinggele Caiyin; Jianjun Qiao

[*] E-mail: m.huang@qub.ac.uk; qinggele@tju.edu.cn; jianjunq@tju.edu.cn

**Contents**


**Table S1**. The coding sequences of yeast codon-optimized genes....................................................3

**Table S2**. Overview of plasmids used in this study..............................................................................9

**Table S3**. Strains used in this study. .....................................................................................................11

**Table S4**. Primers used in this study......................................................................................................14

**Table S5.** The yields of *β*-farnesene of AaFS variants.......................................................................19

**Table S6.** Protein sequences of farnesene synthases used for web logo analysis. ..........................20

**Figure S1.** Comparison of models from three modelling methods ..................................................22

**Figure S2.** Binding environment of FPP. ..........................................................................................23

**Figure S3.** Weblogo plot based on multiple sequence alignment ......................................................24

**Figure S4.** Electropherogram of the SDS-PAGE. ................................................................................25

**Figure S5.** The propensity in amino acid residues in the sequences of AaFS and its 37 orthologues. ..................................................................................................................................................26

**Figure S6.** Site-directed mutagenesis of AaFS ...................................................................................27

**Figure S7.** Product identification and farnesene production of AaFS W299C variant......................28

**Figure S8.** Conformational difference of the farnesyl cation substrate between Fsso-WT and Fsso-C293W. .................................................................................................................................29

**Figure S9.** The substrate binding poses in AaFS WT and variants.. ..................................................30

**Figure S10.** AaFS-F330A MS spectra corresponding to the sesquiterpene peaks. ...........................31

**Table S1**. The coding sequences of yeast codon-optimized genes

| Gene name | Encoding sequences |
|---|---|
| Yeast codon-optimised ***AaFS*** | ATGTCTACTTTGCCAATTTCTTCTGTTTCTTTTTCTTCATCT<br>ACTTCTCCATTGGTCGTTGACGATAAAGTTTCTACAAAACC<br>TGATGTTATTAGACATACTATGAATTTTAATGCTTCTATTT<br>GGGGTGATCAATTTTTGACTTATGATGAACCTGAAGATTT<br>GGTTATGAAAAAACAATTGGTTGAGGAATTGAAAGAGGA<br>AGTTAAAAAAGAGTTAATTACTATTAAAGGTTCAAACGAA<br>CCTATGCAACATGTTAAATTGATTGAATTGATTGATGCTGT<br>TCAAAGATTGGGTATTGCTTATCATTTTGAAGAGGAAATT<br>GAAGAGGCTTTGCAACATATTCATGTTACTTATGGTGAAC<br>AATGGGTTGATAAAGAAAATTTGCAATCTATTTCTTTGTGG<br>TTTAGATTGTTAAGGCAACAAGGTTTTAATGTCTCTTCTGG<br>TGTTTTCAAGGATTTTATGGATGAAAAAGGTAAATTTAAA<br>GAATCTTTGTGTAATGATGCTCAAGGTATTTTGGCTTTGTA<br>TGAAGCTGCTTTTATGAGAGTTGAAGATGAAACTATTTTG<br>GATAATGCTTTGGAGTTCACTAAAGTTCATTTGGATATTAT<br>TGCTAAAGATCCATCTTGTGATTCTTCTTTGAGAACTCAAA<br>TTCATCAAGCATTGAAACAACCATTGAGAAGGAGATTGGC<br>TAGAATTGAGGCTTTGCATTATATGCCAATTTATCAACAA<br>GAAACTTCTCATGATGAAGTTTTGTTGAAATTGGCAAAGT<br>TGGATTTTTCTGTTTTGCAATCTATGCACAAAAAGGAATTG<br>TCTCATATTTGTAAATGGTGGAAAGATTTGGATTTGCAAA<br>ATAAATTGCCATATGTTAGAGATAGAGTTGTTGAAGGTTA<br>TTTTTGGATTTTGTCTATTTATTATGAACCACAACATGCTA<br>GAACTAGAATGTTTTTGATGAAAACTTGTATGTGGTTGGTT<br>GTTTTGGATGATACTTTTGATAATTATGGTACTTATGAAGA<br>ATTGGAAATTTTTACTCAAGCTGTTGAAAGATGGTCTATTT<br>CTTGCTTAGACATGTTGCCTGAGTACATGAAATTGATTTAC<br>CAAGAATTAGTTAATTTGCATGTTGAGATGGAAGAATCTT<br>TGGAAAAAGAAGGTAAGACTTATCAGATTCATTATGTTAA<br>GGAAATGGCTAAAGAATTGGTTAGAAATTATTTGGTTGAA<br>GCTAGATGGTTGAAAGAAGGTTATATGCCAACTTTGGAAG<br>AATATATGTCTGTTTCTATGGTTACTGGTACTTATGGTTTG<br>ATGATTGCTAGATCTTATGTTGGTAGAGGTGATATTGTTAC<br>TGAAGATACTTTTAAATGGGTTTCTTCATATCCACCAATTA |

| | |
|---|---|
| | TTAAGGCTTCTTGTGTTATCGTCAGATTGATGGATGATATT<br>GTTTCTCATAAAGAAGAACAAGAAAGAGGTCATGTTGCTT<br>CTTCTATTGAATGTTATTCTAAAGAATCTGGTGCTTCTGAA<br>GAGGAGGCTTGTGAATATATTTCAAGAAAAGTTGAAGATG<br>CTTGGAAAGTTATTAATAGAGAATCTTTGAGACCAACTGC<br>TGTTCCATTTCCATTGTTGATGCCTGCTATTAATTTGGCTA<br>GAATGTGTGAAGTTTTGTATTCTGTTAATGATGGTTTTACT<br>CATGCTGAAGGTGATATGAAATCTTATATGAAATCATTTTT<br>TGTTCATCCAATGGTTGTTTAA |
| Yeast codon-optimised ***Fsso*** | ATGAATCATTCTTATGCTAATCAATCTGCTCAAGAAGTTAA<br>TATCGTCACAGAGGATACAAGGAGATCAGCTAATTATAAA<br>CCTAACATTTGGAAATACGACTTTTTACAATCATTAGATTC<br>TAAGTACGATGAAGAGGAATTTGTTATGCAGTTGAACAAA<br>AGGGTTACTGAAGTTAAAGGTTTGTTTGTTCAAGAGGCTT<br>CTGTTTTGCAAAAATTGGAATTGGCTGATTGGATTCAAAA<br>ATTGGGTTTGGCTAATTATTTTCAGAAAGACATTAATGAAT<br>TTTTAGAGTCTATTTTAGTCTATGTTAAGAACTCTAATATC<br>AATCCATCTATTGAACATTCTTTGCATGTTTCTGCTTTGTGT<br>TTTAGATTGTTGAGACAACATGGTTATCCTGTTTTACCTGA<br>TACTTTGTCTAACTTTTTGGATGAAAAGGGTAAAGTTATTA<br>GAAAATCTTCATACGTTTGTTATGGTAAAGATGTTGTTGAA<br>TTGTTGGAGGCTTCTCATTTGTCTTTGGAAGGAGAGAAAA<br>TTTTGGATGAGGCTAAAAATTGTGCTATTAATTCTTTGAAA<br>TTTGGTTTTTCACCATCATCAATCAATATCAATAGACATTC<br>AAATTTAGTTGTCGAAAAGATGGTTCATGCTTTGGAATTG<br>CCATCTCATTGGAGAGTTCAGTGGTTTGAAGTTAAATGGC<br>ACGTTGAACAATACAAACAACAAAAGAACGTTGATCCAAT<br>CTTATTGGAATTAACAAAATTAAATTTTAACATGATTCAA<br>GCAAAATTGCAAATTGAAGTTAAAGATTTGTCTAGATGGT<br>GGGAAAATTTGGGTATTAAAAAAGAATTGTCTTTTGCTAG<br>AAATAGATTGGTTGAATCTTTTATGTGTGCTGCTGGTGTCG<br>CATTTGAACCAAAATATAAGGCTGTTAGAAAATGGTTGAC<br>AAAAGTTATTATCTTTGTTTTAATCATCGATGATGTTTATG<br>ATATCCATGCTTCTTTTGAAGAATTGAAACCATTTACTTTG<br>GCTTTTGAAAGATGGGATGATAAAGAATTGGAAGAATTGC<br>CACAGTATATGAAAATTTGTGTCCATGCATTGAAAGATGT |

| | |
|---|---|
| | TACAAATGAAATCGCATATGAAATCGGTGGTGAAAATAAT<br>TTTCATTCAGTCTTACCATATTTGAAAAAGGCTTGGATTGA<br>TTTTTGTAAGGCTTTGTATGTTGAAGCTAAGTGGTACAATA<br>AAGGTTATATTCCATCTTTGGAAGAATATTTGTCTAATGCT<br>TGGATTTCTTCATCTGGTCCTGTTATTTTGTTATTGTCTTAT<br>TTTGCTACTATGAACCAAGCTATGGACATCGATGATTTTTT<br>GCATACATATGAGGATTTAGTTTATAACGTCTCTTTGATTA<br>TTAGATTGTGTAATGATTTGGGTACTACTGCTGCTGAAAG<br>AGAAAAAGGTGATGTTGCTTCTTCTATTTTGTGTTATATGA<br>ACCAAAAAGATGCTTCTGAAGAAAAAGCTAGAAAACATA<br>TTCAAGATATGATCCACAAGGCTTGGAAAAAAATCAATGG<br>TCATTATTGTTCTAATAGAGTTGCTTCTGTTGAACCTTTTTT<br>GACTCAAGCTATTAATGCTGCTAGAGTTGCTCATACTTTGT<br>ATCAAAACGGAGATGGTTTTGGTATTCAAGATAGAGATAT<br>TAAAAAGCATATTTTGTCTTTGGTCGTCGAACCATTGAGAT<br>AA |
| *E. coli* codon-optimised ***AaFS*** | ATGAGCACCCTGCCGATTAGCAGCGTGAGCTTTAGCAGTA<br>GCACGAGCCCGCTGGTGGTGGATGATAAAGTGAGCACCA<br>AACCGGATGTGATTCGCCATACCATGAACTTTAACGCGAG<br>CATTTGGGGCGATCAGTTTCTGACCTATGATGAACCGGAA<br>GATCTGGTGATGAAAAAACAGCTGGTGGAAGAACTGAAA<br>GAAGAAGTGAAGAAGGAATTAATTACCATTAAAGGCAGC<br>AACGAACCGATGCAGCATGTGAAACTGATTGAACTGATTG<br>ATGCGGTGCAGCGCCTGGGCATTGCGTATCATTTTGAAGA<br>GGAAATTGAAGAAGCGCTGCAGCATATTCATGTGACCTAT<br>GGCGAACAGTGGGTGGATAAAGAAAACCTGCAGAGCATT<br>AGCCTGTGGTTTCGCCTGCTGCGTCAGCAAGGCTTTAACGT<br>GAGCAGCGGCGTGTTTAAAGATTTTATGGATGAAAAAGGC<br>AAATTTAAAGAAAGCCTGTGCAACGATGCGCAAGGCATTC<br>TGGCGCTGTATGAAGCGGCGTTTATGCGCGTGGAAGATGA<br>AACCATTCTGGATAACGCGCTGGAATTTACCAAAGTGCAT<br>CTGGATATTATTGCGAAAGATCCGAGCTGCGATAGCAGCC<br>TGCGCACGCAGATTCATCAAGCGCTGAAACAGCCGCTGCG<br>CCGTCGCTTAGCGCGCATTGAAGCGCTGCATTATATGCCG<br>ATTTATCAGCAAGAAACGAGCCATGATGAAGTGCTGCTGA<br>AACTGGCGAAACTGGATTTTAGCGTGCTGCAGAGCATGCA |

| | |
|---|---|
| | TAAAAAAGAACTGAGCCATATTTGCAAATGGTGGAAAGAT<br>CTGGATCTGCAGAACAAACTGCCGTATGTGCGCGATCGCG<br>TGGTGGAAGGCTATTTTTGGATTCTGAGCATTTATTATGAA<br>CCGCAGCATGCGCGCACCCGCATGTTTCTGATGAAAACCT<br>GCATGTGGCTGGTTGTGCTGGATGATACCTTTGATAACTAT<br>GGCACCTATGAAGAACTGGAAATTTTTACCCAAGCGGTGG<br>AACGCTGGAGCATTAGCTGCCTGGATATGCTGCCGGAATA<br>TATGAAACTGATTTATCAAGAACTGGTGAACCTGCATGTG<br>GAGATGGAAGAAAGCCTGGAAAAAGAAGGCAAAACCTAT<br>CAGATTCATTATGTGAAAGAAATGGCGAAAGAACTGGTGC<br>GCAACTATCTGGTGGAAGCGCGCTGGCTGAAAGAAGGCTA<br>TATGCCGACCCTGGAAGAATATATGAGCGTGAGCATGGTG<br>ACCGGCACCTATGGCCTGATGATTGCGCGCAGCTATGTGG<br>GCCGCGGCGATATTGTGACCGAAGATACCTTTAAATGGGT<br>GAGCAGCTATCCGCCGATTATTAAAGCGAGCTGCGTGATT<br>GTGCGCCTGATGGATGATATTGTGAGCCATAAAGAAGAAC<br>AAGAACGCGGCCATGTGGCGAGCAGCATTGAATGCTATAG<br>CAAAGAAAGCGGCGCGAGCGAAGAGGAAGCGTGCGAATA<br>TATTAGCCGCAAAGTGGAAGATGCGTGGAAAGTGATTAAC<br>CGCGAAAGCCTGCGCCCGACCGCGGTGCCGTTTCCGCTGC<br>TGATGCCGGCGATTAACTTAGCGCGCATGTGTGAAGTGCT<br>GTATAGCGTGAACGATGGCTTTACCCATGCGGAAGGCGAT<br>ATGAAAAGTTACATGAAGAGCTTTTTTGTGCATCCGATGG<br>TGGTGTAA |
| *E. coli* codon-optimised ***Fsso*** | ATGAACCATAGCTATGCGAATCAGAGCGCGCAAGAAGTG<br>AACATTGTGACCGAAGATACCCGCCGCAGCGCGAACTATA<br>AACCGAACATTTGGAAATATGACTTCCTGCAGAGTCTGGA<br>TAGCAAATATGATGAAGAGGAATTTGTGATGCAGCTGAAC<br>AAACGCGTGACCGAAGTGAAAGGCCTGTTTGTGCAAGAA<br>GCGAGCGTGCTGCAGAAACTGGAACTGGCGGATTGGATTC<br>AGAAACTGGGCCTGGCGAACTATTTTCAGAAAGATATTAA<br>CGAATTTCTGGAAAGCATTCTGGTGTATGTGAAAAACAGC<br>AACATTAACCCGAGCATTGAACATAGCCTGCATGTGAGCG<br>CGCTGTGCTTTCGCCTGCTGCGTCAGCATGGCTATCCGGTG<br>CTGCCGGATACCCTGAGCAACTTTCTGGATGAAAAAGGCA<br>AAGTGATTCGCAAAAGCAGCTATGTGTGCTATGGCAAAGA |

| | |
|---|---|
| | TGTGGTGGAACTGCTGGAAGCGAGCCATCTGAGCCTGGAA<br>GGCGAAAAAATTCTGGATGAAGCGAAAAACTGCGCGATT<br>AACAGCCTGAAATTTGGCTTTAGCCCGAGCAGCATTAACA<br>TTAACCGCCATAGCAACTTAGTGGTGGAAAAAATGGTGCA<br>TGCCTTAGAATTACCGAGCCATTGGCGCGTGCAGTGGTTT<br>GAAGTGAAATGGCATGTGGAACAGTATAAACAGCAGAAA<br>AACGTGGATCCGATTCTGCTGGAACTGACCAAACTGAACT<br>TTAACATGATTCAAGCGAAACTGCAGATTGAAGTGAAAGA<br>TCTGAGCCGCTGGTGGGAAAACCTGGGCATTAAAAAAGA<br>ACTGAGCTTTGCGCGCAACCGCCTGGTGGAAAGCTTTATG<br>TGCGCGGCGGGCGTGGCGTTTGAGCCAAAATATAAAGCGG<br>TGCGCAAATGGCTGACCAAAGTGATTATTTTTGTGCTGATT<br>ATTGATGATGTGTATGATATTCATGCGAGCTTTGAAGAAC<br>TGAAACCGTTTACCCTGGCGTTTGAACGCTGGGATGATAA<br>AGAACTGGAAGAACTGCCGCAGTATATGAAAATTTGCGTG<br>CATGCGTTAAAGGATGTGACCAACGAAATTGCGTATGAAA<br>TTGGCGGCGAAAACAACTTTCATAGCGTGCTGCCGTATCT<br>GAAAAAAGCGTGGATTGATTTTTGCAAAGCGCTGTATGTG<br>GAAGCGAAATGGTATAACAAAGGCTATATTCCGAGCCTGG<br>AAGAATATCTGAGCAACGCGTGGATTAGCAGTAGCGGCCC<br>GGTGATTCTGTTACTGAGCTATTTTGCGACCATGAACCAA<br>GCGATGGATATTGACGATTTTCTGCACACCTATGAAGATC<br>TGGTGTATAACGTGAGCCTGATTATTCGCCTGTGCAACGA<br>TCTGGGCACCACCGCGGCGGAACGCGAAAAAGGCGATGT<br>GGCGAGCAGCATTCTGTGCTATATGAATCAGAAAGATGCG<br>AGCGAAGAAAAAGCGCGCAAACATATTCAAGATATGATT<br>CATAAAGCGTGGAAAAAAATTAACGGCCATTATTGCAGCA<br>ACCGCGTGGCGAGCGTGGAACCGTTTCTGACCCAAGCGAT<br>TAACGCGGCGCGCGTGGCGCATACCCTGTATCAGAACGGC<br>GATGGCTTTGGCATTCAAGATCGCGATATTAAAAAACATA<br>TTCTGAGCCTGGTGGTTGAACCGCTGCGCTAA |

**Table S2**. Overview of plasmids used in this study.

| Plasmid | Description | Source |
|---|---|---|
| pESC-*Fsso* | pESC-URA vector containing yeast codon-optimized *Fsso* | this study |
| pESC-*AaFS* | pESC-URA vector containing yeast codon-optimized *AaFS* | this study |
| pESC-*AaFS*-D471N | pESC-URA vector containing *AaFS* $^{D471N}$ | this study |
| pESC-*AaFS*-E405A | pESC-URA vector containing *AaFS* $^{E405A}$ | this study |
| pESC-*AaFS*-R468A | pESC-URA vector containing *AaFS* $^{R468A}$ | this study |
| pESC-*AaFS*-Y402L | pESC-URA vector containing *AaFS* $^{Y402L}$ | this study |
| pESC-*AaFS*-Y402F | pESC-URA vector containing *AaFS* $^{Y402F}$ | this study |
| pESC-*AaFS*-V324A | pESC-URA vector containing *AaFS* $^{V324A}$ | this study |
| pESC-*AaFS*-Y430A | pESC-URA vector containing *AaFS* $^{Y430A}$ | this study |
| pESC-*AaFS*-M433A | pESC-URA vector containing *AaFS* $^{M433A}$ | this study |
| pESC-*AaFS*-L539A | pESC-URA vector containing *AaFS* $^{L539A}$ | this study |
| pESC-*AaFS*-L323A | pESC-URA vector containing *AaFS* $^{L323A}$ | this study |
| pESC-*AaFS*-F330A | pESC-URA vector containing *AaFS* $^{F330A}$ | this study |
| pESC-*AaFS*-T427A | pESC-URA vector containing *AaFS* $^{T427A}$ | this study |
| pESC-*AaFS*-V467A | pESC-URA vector containing *AaFS* $^{V467A}$ | this study |
| pESC-*AaFS*-F553A | pESC-URA vector containing *AaFS* $^{F553A}$ | this study |
| pESC-*AaFS*-V324L | pESC-URA vector containing *AaFS* $^{V324L}$ | this study |
| pESC-*AaFS*-V324F | pESC-URA vector containing *AaFS* $^{V324F}$ | this study |
| pESC-*AaFS*-M433L | pESC-URA vector containing *AaFS* $^{M433L}$ | this study |
| pESC-*AaFS*-M433I | pESC-URA vector containing *AaFS* $^{M433I}$ | this study |
| pESC-*AaFS*-L539Y | pESC-URA vector containing *AaFS* $^{L539Y}$ | this study |
| pESC-*AaFS*-L326I | pESC-URA vector containing *AaFS* $^{L326I}$ | this study |
| pESC-*AaFS*-F330Y | pESC-URA vector containing *AaFS* $^{F330Y}$ | this study |
| pESC-*AaFS*-F330T | pESC-URA vector containing *AaFS* $^{F330T}$ | this study |
| pESC-*AaFS*-L398F | pESC-URA vector containing *AaFS* $^{L398F}$ | this study |
| pESC-*AaFS*-L398Y | pESC-URA vector containing *AaFS* $^{L398Y}$ | this study |
| pESC-*AaFS*-T429S | pESC-URA vector containing *AaFS* $^{T429S}$ | this study |
| pESC-*AaFS*-T429V | pESC-URA vector containing *AaFS* $^{T429V}$ | this study |
| pESC-*AaFS*-H33R | pESC-URA vector containing *AaFS* $^{H33R}$ | this study |
| pESC-*AaFS*-A39P | pESC-URA vector containing *AaFS* $^{A39P}$ | this study |
| pESC-*AaFS*-I89L | pESC-URA vector containing *AaFS* $^{I89L}$ | this study |
| pESC-*AaFS*-Q130Y | pESC-URA vector containing *AaFS* $^{Q130Y}$ | this study |

| | | |
|---|---|---|
| pESC-*AaFS*-Q142H | pESC-URA vector containing *AaFS* $^{Q142H}$ | this study |
| pESC-*AaFS*-G149D | pESC-URA vector containing *AaFS* $^{G149D}$ | this study |
| pESC-*AaFS*-S260N | pESC-URA vector containing *AaFS* $^{S260N}$ | this study |
| pESC-*AaFS*-Y288F | pESC-URA vector containing *AaFS* $^{Y288F}$ | this study |
| pESC-*AaFS*-S350N | pESC-URA vector containing *AaFS* $^{S350N}$ | this study |
| pESC-*AaFS*-R407K | pESC-URA vector containing *AaFS* $^{R407K}$ | this study |
| pESC-*AaFS*-M421L | pESC-URA vector containing *AaFS* $^{M421L}$ | this study |
| pESC-*AaFS*-V423N | pESC-URA vector containing *AaFS* $^{V423N}$ | this study |
| pESC-*AaFS*-E478A | pESC-URA vector containing *AaFS* $^{E478A}$ | this study |
| pESC-*AaFS*-Y402A | pESC-URA vector containing *AaFS* $^{Y402A}$ | this study |
| pESC-*AaFS*-Y402V | pESC-URA vector containing *AaFS* $^{Y402V}$ | this study |
| pESC-*AaFS*-Y402I | pESC-URA vector containing *AaFS* $^{Y402I}$ | this study |
| pESC-*AaFS*-Y402W | pESC-URA vector containing *AaFS* $^{Y402W}$ | this study |
| pESC-*AaFS*-Y402T | pESC-URA vector containing *AaFS* $^{Y402T}$ | this study |
| pESC-*AaFS*-Y402M | pESC-URA vector containing *AaFS* $^{Y402M}$ | this study |
| pESC-*AaFS*-Y402Q | pESC-URA vector containing *AaFS* $^{Y402Q}$ | this study |
| pESC-*AaFS*-Y402H | pESC-URA vector containing *AaFS* $^{Y402H}$ | this study |
| pESC-*AaFS*-W299C | pESC-URA vector containing *AaFS* $^{W299C}$ | this study |
| pESC-*AaFS*-W299G | pESC-URA vector containing *AaFS* $^{W299G}$ | this study |
| pESC-*AaFS*-W299A | pESC-URA vector containing *AaFS* $^{W299A}$ | this study |
| pESC-*AaFS*-W299L | pESC-URA vector containing *AaFS* $^{W299L}$ | this study |
| pESC-*AaFS*-W299S | pESC-URA vector containing *AaFS* $^{W299S}$ | this study |
| pESC-*AaFS*-W299F | pESC-URA vector containing *AaFS* $^{W299F}$ | this study |
| pESC-*AaFS*-W299M | pESC-URA vector containing *AaFS* $^{W299M}$ | this study |
| pESC-*AaFS*-W299K | pESC-URA vector containing *AaFS* $^{W299K}$ | this study |
| pESC-*AaFS*-W299D | pESC-URA vector containing *AaFS* $^{W299D}$ | this study |
| pESC-*Fsso*-C293W | pESC-URA vector containing *Fsso* $^{C293W}$ | this study |
| pET28a | pBR322 ori, $Kan^R$ | stored in the lab |
| pET28a-AaFS | pET28a, $P_{T7}$-*His$_6$*-*AaFS* | this study |
| pET28a-Fsso | pET28a, $P_{T7}$-*His$_6$*-*Fsso* | this study |
| pET28a-AaFS-Q142H | pET28a, $P_{T7}$-*His$_6$*-*AaFS* $^{Q142H}$ | this study |
| pET28a-AaFS-L326I | pET28a, $P_{T7}$-*His$_6$*-*AaFS* $^{L326I}$ | this study |
| pET28a-AaFS-M433I | pET28a, $P_{T7}$-*His$_6$*-*AaFS* $^{M433I}$ | this study |

**Table S3**. Strains used in this study.

| Strain | Description | Source |
|---|---|---|
| Sc027 | CEN.PK2-1C derivative; *leu2-3112:: G418R_*$T_{ERG1}$*9-ERG19-*$P_{GAL1}$*_*$P_{GAL10}$*-ERG8-*$T_{ERG8}$*; his3Δ1:: HIS3_*$T_{ERG12}$*-ERG12-*$P_{GAL1}$*_*$P_{GAL10}$*-ERG10-*$T_{ERG10}$*; ade1Δ::* $T_{HMG1}$*-tHMG1-*$P_{GAL1}$*_*$P_{GAL10}$*-IDI1-*$T_{IDI1}$*_ADE1; ura3-52::* $T_{HMG1}$*-tHMG1-*$P_{GAL1}$*_*$P_{GAL10}$*-ERG13-*$T_{ERG13}$*; trp1-289::* $T_{HMG1}$*-tHMG1-*$P_{GAL1}$*_*$P_{GAL10}$*-ERG20-*$T_{ERG20}$*_TRP1*; | stored in the lab |
| Sc027-Fsso | Expressing pESC-*Fsso* in Sc027 | this study |
| Sc027-AaFS | Expressing pESC-*AaFS* in Sc027 | this study |
| Sc027-AaFS-D471N | Expressing pESC-*AaFS* $^{D471N}$ in Sc027 | this study |
| Sc027-AaFS-E405A | Expressing pESC-*AaFS* $^{E405A}$ in Sc027 | this study |
| Sc027-AaFS-R468A | Expressing pESC-*AaFS* $^{R468A}$ in Sc027 | this study |
| Sc027-AaFS-Y402L | Expressing pESC-*AaFS* $^{\mathrm{Y402L}}$ in Sc027 | this study |
| Sc027-AaFS-Y402F | Expressing pESC-*AaFS* $^{\mathrm{Y402F}}$ in Sc027 | this study |
| Sc027-AaFS-V324A | Expressing pESC-*AaFS* $^{\mathrm{V324A}}$ in Sc027 | this study |
| Sc027-AaFS-Y430A | Expressing pESC-*AaFS* $^{\mathrm{Y430A}}$ in Sc027 | this study |
| Sc027-AaFS-M433A | Expressing pESC-*AaFS* $^{\mathrm{M433A}}$ in Sc027 | this study |
| Sc027-AaFS-L539A | Expressing pESC-*AaFS* $^{\mathrm{L539A}}$ in Sc027 | this study |
| Sc027-AaFS-L323A | Expressing pESC-*AaFS* $^{\mathrm{L323A}}$ in Sc027 | this study |
| Sc027-AaFS-F330A | Expressing pESC-*AaFS* $^{\mathrm{F330A}}$ in Sc027 | this study |
| Sc027-AaFS-T427A | Expressing pESC-*AaFS* $^{\mathrm{T427A}}$ in Sc027 | this study |
| Sc027-AaFS-V467A | Expressing pESC-*AaFS* $^{\mathrm{V467A}}$ in Sc027 | this study |
| Sc027-AaFS-F553A | Expressing pESC-*AaFS* $^{\mathrm{F553A}}$ in Sc027 | this study |
| Sc027-AaFS-V324L | Expressing pESC-*AaFS* $^{\mathrm{V324L}}$ in Sc027 | this study |
| Sc027-AaFS-V324F | Expressing pESC-*AaFS* $^{\mathrm{V324F}}$ in Sc027 | this study |
| Sc027-AaFS-M433L | Expressing pESC-*AaFS* $^{\mathrm{M433L}}$ in Sc027 | this study |
| Sc027-AaFS-M433I | Expressing pESC-*AaFS* $^{\mathrm{M433I}}$ in Sc027 | this study |
| Sc027-AaFS-L539Y | Expressing pESC-*AaFS* $^{\mathrm{L539Y}}$ in Sc027 | this study |
| Sc027-AaFS-L326I | Expressing pESC-*AaFS* $^{\mathrm{L326I}}$ in Sc027 | this study |
| Sc027-AaFS-F330Y | Expressing pESC-*AaFS* $^{\mathrm{F330Y}}$ in Sc027 | this study |
| Sc027-AaFS-F330T | Expressing pESC-*AaFS* $^{\mathrm{F330T}}$ in Sc027 | this study |
| Sc027-AaFS-L398F | Expressing pESC-*AaFS* $^{\mathrm{L398F}}$ in Sc027 | this study |
| Sc027-AaFS-L398Y | Expressing pESC-*AaFS* $^{\mathrm{L398Y}}$ in Sc027 | this study |
| Sc027-AaFS-T429S | Expressing pESC-*AaFS* $^{\mathrm{T429S}}$ in Sc027 | this study |

| | | |
|---|---|---|
| Sc027-AaFS-T429V | Expressing pESC-*AaFS* $^{T429V}$ in Sc027 | this study |
| Sc027-AaFS-H33R | Expressing pESC-*AaFS* $^{H33R}$ in Sc027 | this study |
| Sc027-AaFS-A39P | Expressing pESC-*AaFS* $^{A39P}$ in Sc027 | this study |
| Sc027-AaFS-I89L | Expressing pESC-*AaFS* $^{I89L}$ in Sc027 | this study |
| Sc027-AaFS-Q130Y | Expressing pESC-*AaFS* $^{Q130Y}$ in Sc027 | this study |
| Sc027-AaFS-Q142H | Expressing pESC-*AaFS* $^{Q142H}$ in Sc027 | this study |
| Sc027-AaFS-G149D | Expressing pESC-*AaFS* $^{G149D}$ in Sc027 | this study |
| Sc027-AaFS-S260N | Expressing pESC-*AaFS* $^{S260N}$ in Sc027 | this study |
| Sc027-AaFS-Y288F | Expressing pESC-*AaFS* $^{Y288F}$ in Sc027 | this study |
| Sc027-AaFS-S350N | Expressing pESC-*AaFS* $^{S350N}$ in Sc027 | this study |
| Sc027-AaFS-R407K | Expressing pESC-*AaFS* $^{R407K}$ in Sc027 | this study |
| Sc027-AaFS-M421L | Expressing pESC-*AaFS* $^{M421L}$ in Sc027 | this study |
| Sc027-AaFS-V423N | Expressing pESC-*AaFS* $^{V423N}$ in Sc027 | this study |
| Sc027-AaFS-E478A | Expressing pESC-*AaFS* $^{E478A}$ in Sc027 | this study |
| Sc027-AaFS-Y402A | Expressing pESC-*AaFS* $^{Y402A}$ in Sc027 | this study |
| Sc027-AaFS-Y402V | Expressing pESC-*AaFS* $^{Y402V}$ in Sc027 | this study |
| Sc027-AaFS-Y402I | Expressing pESC-*AaFS* $^{Y402I}$ in Sc027 | this study |
| Sc027-AaFS-Y402W | Expressing pESC-*AaFS* $^{Y402W}$ in Sc027 | this study |
| Sc027-AaFS-Y402T | Expressing pESC-*AaFS* $^{Y402T}$ in Sc027 | this study |
| Sc027-AaFS-Y402M | Expressing pESC-*AaFS* $^{Y402M}$ in Sc027 | this study |
| Sc027-AaFS-Y402Q | Expressing pESC-*AaFS* $^{Y402Q}$ in Sc027 | this study |
| Sc027-AaFS-Y402H | Expressing pESC-*AaFS* $^{Y402H}$ in Sc027 | this study |
| Sc027-AaFS-W299C | Expressing pESC-*AaFS* $^{W299C}$ in Sc027 | this study |
| Sc027-AaFS-W299G | Expressing pESC-*AaFS* $^{W299G}$ in Sc027 | this study |
| Sc027-AaFS-W299A | Expressing pESC-*AaFS* $^{W299A}$ in Sc027 | this study |
| Sc027-AaFS-W299L | Expressing pESC-*AaFS* $^{W299L}$ in Sc027 | this study |
| Sc027-AaFS-W299S | Expressing pESC-*AaFS* $^{W299S}$ in Sc027 | this study |
| Sc027-AaFS-W299F | Expressing pESC-*AaFS* $^{W299F}$ in Sc027 | this study |
| Sc027-AaFS-W299M | Expressing pESC-*AaFS* $^{W299M}$ in Sc027 | this study |
| Sc027-AaFS-W299K | Expressing pESC-*AaFS* $^{W299K}$ in Sc027 | this study |
| Sc027-AaFS-W299D | Expressing pESC-*AaFS* $^{W299D}$ in Sc027 | this study |
| Sc027-Fsso-C293W | Expressing pESC-*Fsso* $^{C293W}$ in Sc027 | this study |
| BL21 | BL21 (DE3) | stored in the lab |
| BL21-pET28a-AaFS | Expressing pET28a-AaFS in BL21 | this study |
| BL21- pET28a-Fsso | Expressing pET28a-Fsso in BL21 | this study |

| | | |
|---|---|---|
| BL21- pET28a-AaFS-Q142H | Expressing pET28a-AaFS $^{Q142H}$ in BL21 | this study |
| BL21- pET28a-AaFS-L326I | Expressing p pET28a-AaFS $^{L326I}$ in BL21 | this study |
| BL21- pET28a-AaFS-M433I | Expressing pET28a-AaFS $^{M433I}$ in Sc027 | this study |

**Table S4**. Primers used in this study.

| Name | Primer sequence (5'-3') |
|---|---|
| | Site-directed mutagenesis of *AaFS for expression in yeast* |
| AaFS-F | TAACGTCAAGGAGAAAAAACCCCATGTCTACTTTGCCAATTTCTT |
| AaFS-R | GATCTTAGCTAGCCGCGGTACCTTAAACAACCATTGGATGAACAA |
| D471N-F | TATCGTCAGATTGATGAATGATATTGTTTC |
| D471N-R | GAAACAATATCATTCATCAATCTGACGATA |
| E405A-F | GAAATTATTTGGTTGCTGCTAGATGGTTGAAA |
| E405A-R | TTTCAACCATCTAGCAGCAACCAAATAATTTC |
| R468A-F | CTTCTTGTGTTATCGTCGCTTTGATGGATGATATTGT |
| R468A-R | ACAATATCATCCATCAAAGCGACGATAACACAAGAAG |
| Y402L-F | AAAGAATTGGTTAGAAATTTGTTGGTTGCTGCTAG |
| Y402L-R | CTAGCAGCAACCAACAAATTTCTAACCAATTCTTT |
| Y402F-F | AAGAATTGGTTAGAAATTTTTTGGTTGCTGCTAGAT |
| Y402F-R | ATCTAGCAGCAACCAAAAAATTTCTAACCAATTCTT |
| V324A-F | AACTTGTATGTGGTTGGCAGTTTTGGATGATACT |
| V324A-R | AGTATCATCCAAAACTGCCAACCACATACAAGTT |
| Y430A-F | TCTATGGTTACTGGTACTGCTGGTTTGATGATTGCT |
| Y430A-R | AGCAATCATCAAACCAGCAGTACCAGTAACCATAGA |
| M433A-F | TGGTACTTATGGTTTGGCTATTGCTAGATCTTA |
| M433R-R | TAAGATCTAGCAATAGCCAAACCATAAGTACCA |
| L539A-F | GATGCCTGCTATTAATGCTGCTAGAATGTGTGAAG |
| L539A-R | CTTCACACATTCTAGCAGCATTAATAGCAGGCATC |
| L323A-F | GAAAACTTGTATGTGGGCTGCAGTTTTGGATGATACT |
| L323A-R | AGTATCATCCAAAACTGCAGCCCACATACAAGTTTTC |
| F330A-F | GGCAGTTTTGGATGATACTTATGATAATTATGGTACTTAT |
| F330A-R | ATAAGTACCATAATTATCATAAGTATCATCCAAAACTGCC |
| T427A-F | ATGTCTGTTTCTATGGTTGCTGGTACTTATGGTTTGGCT |
| T427A-R | AGCCAAACCATAAGTACCAGCAACCATAGAAACAGACAT |
| V467A-F | TAAGGCTTCTTGTGTTATCGCTGCTTTGATGGATGAT |
| V467A-R | ATCATCCATCAAAGCAGCGATAACACAAGAAGCCTTA |
| F553A-F | TGTATTCTGTTAATGATGGTGCTACTCATGCTGAAGGTGA |
| F553A-R | TCACCTTCAGCATGAGTAGCACCATCATTAACAGAATACA |
| V324L-F | GATGAAAACTTGTATGTGGTTGTTGGTTTTGGATGATACTT |
| V324L-R | AAGTATCATCCAAAACCAACAACCACATACAAGTTTTCATC |
| V324F-F | GAAAACTTGTATGTGGTTGTTTGTTTTGGATGATACT |

| | |
|---|---|
| V324F-R | AGTATCATCCAAAACAAACAACCACATACAAGTTTTC |
| M433L-F | GGTACTTATGGTTTGTTGATTGCTAGATCTTATG |
| M433L-R | CATAAGATCTAGCAATCAACAAACCATAAGTACC |
| M433I-F | CTGGTACTTATGGTTTGATTATTGCTAGATCTTATGT |
| M433I-R | ACATAAGATCTAGCAATAATCAAACCATAAGTACCAG |
| L539Y-F | ATGCCTGCTATTAATTATGCTAGAATGTGTGAAGTTTTGT |
| L539Y-R | ACAAAACTTCACACATTCTAGCATAATTAATAGCAGGCAT |
| L326I-F | GTATGTGGTTGTTTGTTATTGATGATACTTTTGATAATTATGGTAC TT |
| L326I-R | AAGTACCATAATTATCAAAAGTATCATCAATAACAAACAACCACA T |
| F330Y-F | TTTGTTTTGGATGATACTTACGATAATTATGGTACTTATGAA |
| F330Y-R | TTCATAAGTACCATAATTATCGTAAGTATCATCCAAAACAAA |
| F330T-F | TTGTTTTGGATGATACTACTGATAATTATGGTACTTATGAA |
| F330T-R | TTCATAAGTACCATAATTATCAGTAGTATCATCCAAAACAA |
| L398F-F | GGAAATGGCTAAAGAATTTGTTAGAAATTATTTGGTT |
| L398F-R | AACCAAATAATTTCTAACAAATTCTTTAGCCATTTCC |
| L398Y-F | GGAAATGGCTAAAGAATATGTTAGAAATTATTT |
| L398Y-R | AAATAATTTCTAACATATTCTTTAGCCATTTCC |
| T429S-F | CTGTTTCTATGGTTACTGGTTCTTATGGTTTGATTATTGC |
| T429S-R | AATAATCAAACCATAAGAACCAGTAACCATAGAAACA |
| T429V-F | TTTCTATGGTTACTGGTGTTTATGGTTTGATTATTGC |
| T429V-R | GCAATAATCAAACCATAAACACCAGTAACCATAGAAA |
| H33R-F | TGATGTTATTAGAAGAACTATGAATTTTAATGCTTC |
| H33R-R | GAAGCATTAAAATTCATAGTTCTTCTAATAACATCA |
| A39P-F | GACATACTATGAATTTTAATCCTTCTATTTGGGGTGA |
| A39P-R | TCACCCCAAATAGAAGGATTAAAATTCATAGTATGTC |
| I89L-F | CAACATGTTAAATTGTTGGAATTGATTGATGCTGTTCAAAG |
| I89L-R | CTTTGAACAGCATCAATCAATTCCAACAATTTAACATGTTG |
| Q130Y-F | GTTGATAAAGAAAATTTGTATTCTATTTCTTTGTGG |
| Q130Y-R | CCACAAAGAAATAGAATACAAATTTTCTTTATCAAC |
| Q142H-F | AGATTGTTAAGGCAACATGGTTTTAATGTCTCT |
| Q142H-R | AGAGACATTAAAACCATGTTGCCTTAACAATCT |
| G149D-F | GGTTTTAATGTCTCTTCTGATGTTTTCAAGGATTTTATG |
| G149D-R | CATAAAATCCTTGAAAACATCAGAAGAGACATTAAAACC |
| S260N-F | TGGCAAAGTTGGATTTTAATGTTTTGCAATCTATGCAC |

| | |
|---|---|
| S260N-R | GTGCATAGATTGCAAAACATTAAAATCCAACTTTGCCA |
| Y288F-F | ATTTGCAAAATAAATTGCCATTTGTTAGAGATAGAGTTG |
| Y288F-R | CAACTCTATCTCTAACAAATGGCAATTTATTTTGCAAAT |
| S350N-F | TCAAGCTGTTGAAAGATGGAATATTTCTTGCTTAGACA |
| S350N-R | TGTCTAAGCAAGAAATATTCCATCTTTCAACAGCTTGA |
| R407K-F | AAATTATTTGGTTGAAGCTAAATGGTTGAAAGAA |
| R407K-R | TTCTTTCAACCATTTAGCTTCAACCAAATAATTT |
| M421L-F | CAACTTTGGAAGAATATTTGTCTGTTTCTATGGTTA |
| M421L-R | TAACCATAGAAACAGACAAATATTCTTCCAAAGTTG |
| V423N-F | CTTTGGAAGAATATATGTCTAATTCTATGGTTACTGGTGTT |
| V423N-R | AACACCAGTAACCATAGAATTAGACATATATTCTTCCAAAG |
| E478A-F | ATATTGTTTCTCATAAAGCAGAACAAGAAAGAGGTCATGTTGCTTC |
| E478A-R | AGCAACATGACCTCTTTCTTGTTCTGCTTTATGAGAAACAATATCA |
| Y402A-F | AGAATTGGTTAGAAATGCTTTGGTTGAAGCTAGATGG |
| Y402A-R | CCATCTAGCTTCAACCAAAGCATTTCTAACCAATTCT |
| Y402V-F | AGAATTGGTTAGAAATGTTTTGGTTGAAGCTAGAT |
| Y402V-R | ATCTAGCTTCAACCAAAACATTTCTAACCAATTCT |
| Y402I-F | GCTAAAGAATTGGTTAGAAATATTTTGGTTGAAGCTAGATGGT |
| Y402I-R | ACCATCTAGCTTCAACCAAAATATTTCTAACCAATTCTTTAGC |
| Y402W-F | AAAGAATTGGTTAGAAATTGGTTGGTTGAAGCTA |
| Y402W-R | TAGCTTCAACCAACCAATTTCTAACCAATTCTTT |
| Y402T-F | TAAAGAATTGGTTAGAAATACTTTGGTTGAAGCTA |
| Y402T-R | TAGCTTCAACCAAAGTATTTCTAACCAATTCTTTA |
| Y402M-F | AGAATTGGTTAGAAATATGTTGGTTGAAGCTAGATGGT |
| Y402M-R | ACCATCTAGCTTCAACCAACATATTTCTAACCAATTCT |
| Y402Q-F | AAAGAATTGGTTAGAAATCAATTGGTTGAAGCTAGA |
| Y402Q-R | TCTAGCTTCAACCAATTGATTTCTAACCAATTCTTT |
| Y402H-F | AAGAATTGGTTAGAAATCATTTGGTTGAAGCTAG |
| Y402H-R | CTAGCTTCAACCAAATGATTTCTAACCAATTCTT |
| W299G-F | GAGTTGTTGAAGGTTATTTTGCTATTTTGTCTATTTATTATG |
| W299G-R | CATAATAAATAGACAAAATAGCAAAATAACCTTCAACAACTC |
| W299C-F | TGTTGAAGGTTATTTTTGCATTTTGTCTATTTATTATGAACCAC |
| W299C-R | GTGGTTCATAATAAATAGACAAAATGCAAAAATAACCTTCAACA |
| W299A-F | AGTTGTTGAAGGTTATTTTGCTATTTTGTCTATTTATTATGAACCA |

| | |
|---|---|
| W299A-R | TGGTTCATAATAAATAGACAAAATAGCAAAATAACCTTCAACAACT |
| W299L-F | TTGTTGAAGGTTATTTTTTGATTTTGTCTATTTATTATGAACC |
| W299L-R | GGTTCATAATAAATAGACAAAATCAAAAAATAACCTTCAACAA |
| W299S-F | TTGTTGAAGGTTATTTTTCTATTTTGTCTATTTATTATGAAC |
| W299S-R | GTTCATAATAAATAGACAAAATAGAAAAATAACCTTCAACAA |
| W299F-F | GTTGTTGAAGGTTATTTTTTTATTTTGTCTATTTATTATGAACCA |
| W299F-R | TGGTTCATAATAAATAGACAAAATAAAAAAATAACCTTCAACAAC |
| W299M-F | AGAGTTGTTGAAGGTTATTTTATGATTTTGTCTATTTATTATGAACC |
| W299M-R | TGTGGTTCATAATAAATAGACAAAATCATAAAATAACCTTCAACA |
| W299K-F | TGTTGAAGGTTATTTTAAAATTTTGTCTATTTATTATGAACCAC |
| W299K-R | GTGGTTCATAATAAATAGACAAAATTTTAAAATAACCTTCAACA |
| W299D-F | GAGTTGTTGAAGGTTATTTTGATATTTTGTCTATTTATTATGAACCAC |
| W299D-R | GTTGTGGTTCATAATAAATAGACAAAATATCAAAATAACCTTCAAC |
| | <u>Site-directed mutagenesis of *Fsso for expression in yeast*</u> |
| Fsso-F | GTCAAGGAGAAAAAACCCCATGAATCATTCTTATGCTAATCAATC |
| Fsso-R | ATCTTAGCTAGCCGCGGTACCTTATCTCAATGGTTCGACGACC |
| C293W-F | TGGTTGAATCTTTTATGTGGGCTGCTGGTGTCGCATTT |
| C293W-R | AAATGCGACACCAGCAGCCCACATAAAAGATTCAACCA |
| | <u>Site-directed mutagenesis of *AaFS for expression in E. coli*</u> |
| AaFS-F1 | CCGCCATGGCCAGCACCCTGCCGATTAGCAGC |
| AaFS-R1 | CATGCTCGAGCACCACCATCGGATGCACAAAAAAGC |
| Q142H-F1 | TGGTTTCGCCTGCTGCGTCATCAAGGCTTTAACGTGAGC |
| Q142H-R1 | GCTCACGTTAAAGCCTTGATGACGCAGCAGGCGAAACCA |
| L326I-F1 | ATGAAAACCTGCATGTGGATTGTTGTGCTGGATGATACC |
| L326I-R1 | GGTATCATCCAGCACAACAATCCACATGCAGGTTTTCAT |
| M433I-F1 | ACCGGCACCTATGGCCTGATTATTGCGCGCAGCTATG |
| M433I-R1 | CATAGCTGCGCGCAATAATCAGGCCATAGGTGCCGGT |

**Table S5.** The yields of *β*-farnesene of AaFS variants.

| Position | Mutation | Yield (mg/L) |
|---|---|---|
| WT | - | 450.65 |
| | **Mg Coordination** | |
| D471 | D471N | 193.99 |
| S475 | S475T | 627.04 |
| E405 | E405A | 146.02 |
| | **PPi binding** | |
| R468 | R468A | 145.37 |
| | **First sphere** | |
| C320 | C320V | 3.65 |
| L323 | L323A | 21.50 |
| V324 | V324S | 11.34 |
| L326 | L326I | 3877.42 |
| Y402 | Y402F | 244.92 |
| T429 | T429G | 488.15 |
| | T429A | 231.43 |
| | T429S | 443.97 |
| Y430 | Y430A | 254.22 |
| | Y430G | 182.34 |
| | Y430L | 143.32 |
| M433 | M433L | 267.59 |
| | M433I | 2961.13 |
| V467 | V467A | 103.42 |
| | V467I | 767.65 |
| | V467G | 187.64 |
| | V467F | 230.34 |
| L539 | L539F | 148.85 |
| | **Residues surrounding Y402** | |
| L398 | L398A | 125.05 |
| T427 | T427A | 81.20 |
| F330 | F330A | 190.08 |

**Table S6.** Protein sequences of farnesene synthases used for web logo analysis.

| Genes | Accession Number | Sources |
|---|---|---|
| Pt5 | AF543528.1 | *Pinus taeda* |
| CsAFS | GFMV01032657.1 | *Camellia sinensis* |
| RcSeTPS7 | JN315866.1 | *Ricinus communis* |
| Fsso | MT559750 | Soybean |
| CmTPSDul | NM001297455 | *Cucumis melo* |
| MdAFS | AY787633.1 | *Malus domestica* |
| PtTPS2 | JF449451.1 | *Populus trichocarpa* |
| SbTPS3 | XM021464557 | *Sorghum bicolor* |
| CsFS | AY640154.1 | *Cucumis sativus* |
| TPS10-mex | GQ253104.1 | *Zea mays mexicana* |
| TPS10-per | GQ253107.1 | *Zea perennis* |
| TPS10-dip | GQ253106.1 | *Zea diploperennis* |
| AdAFS1 | FJ265785.1 | *Actinidia deliciosa* |
| LoTPS2 | KT963004 | *Lilium* 'Siberia' |
| MxpFAS | AF024615.1 | *Mentha* x *piperita* |
| PcAFS1 | AY566286.1 | *Pyrus communis* |
| OfTPS4 | KT591183 | *Osmanthus fragrans* |
| TPS-Far | AY473627.1 | *Picea abies* |
| MkTPS2 | KX171230.1 | *Murraya koenigii* |
| CjFS | AF374462.1 | *Citrus junos* |
| AjTPS2 | MN654902.1 | *Albizia julibrissin* |
| tps1 | AF529266.1 | *Zea mays* |
| VvCSbOciF | HM807389.1 | *Vitis vinifera* |
| PdTPS6 | MK426624.1 | *Prunus dulcis* |
| ZmTPS10 | GQ253105.1 | *Zea mays* |
| CsSesquiTPS3 | MF280921. | *Citrus sinensis* |
| AFS1 | AY182241.2 | *Malus domestica* |
| TlTPS18983 | MG680745.1 | *Thapsia laciniata* |
| AaFS | AY835398.1 | *Artemisia annua* |
| PmeTPS4 | AY906867.1 | *Pseudotsuga menziesii* |
| VvCSaFar | HM807379.1 | *Vitis vinifera* |
| At3g25810 | BT053763.1 | *Arabidopsis thaliana* |
| Pg×eTPS-Far/Oci | HQ426157.1 | *Picea*spp |
| Mr-Bfs | KM586847.1 | *Matricaria recutita* |

| | | |
|---|---|---|
| MrTPS6 | KJ020283.1 | *Matricaria recutita* |
| CsTPS5FN | KY014560.1 | *Cannabis sativa* |
| PsTPS5 | GU248335.1 | *Pinus sylvestris* |

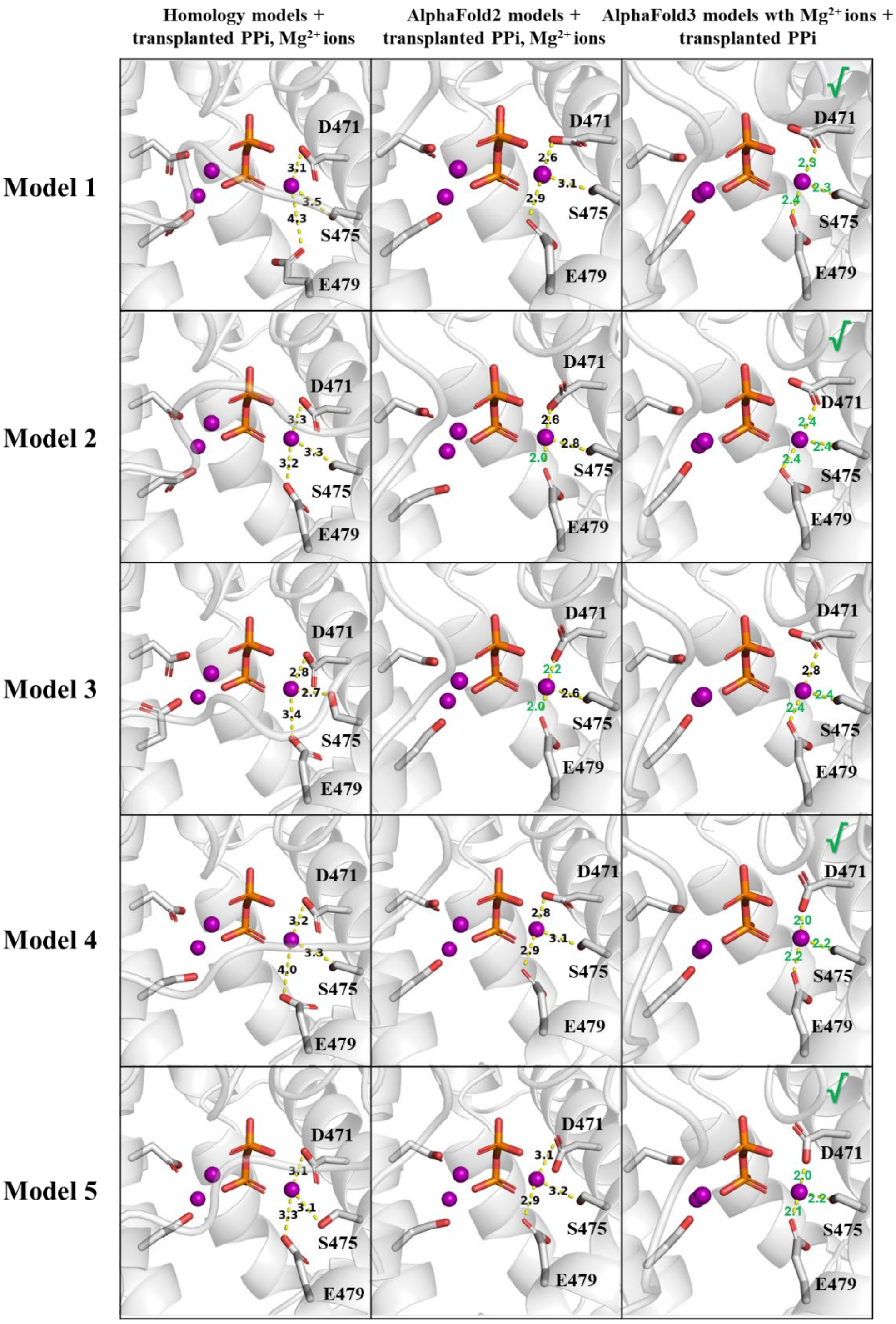


**Figure S1.** Comparison of models from three modelling methods. The key residues located in DDxxD and DSE motifs are shown in stick models. The distances between Mg1 and the three coordinating key residues (D471, S475, E479) were labelled and the distances ≤2.4 Å are highlighted in green.

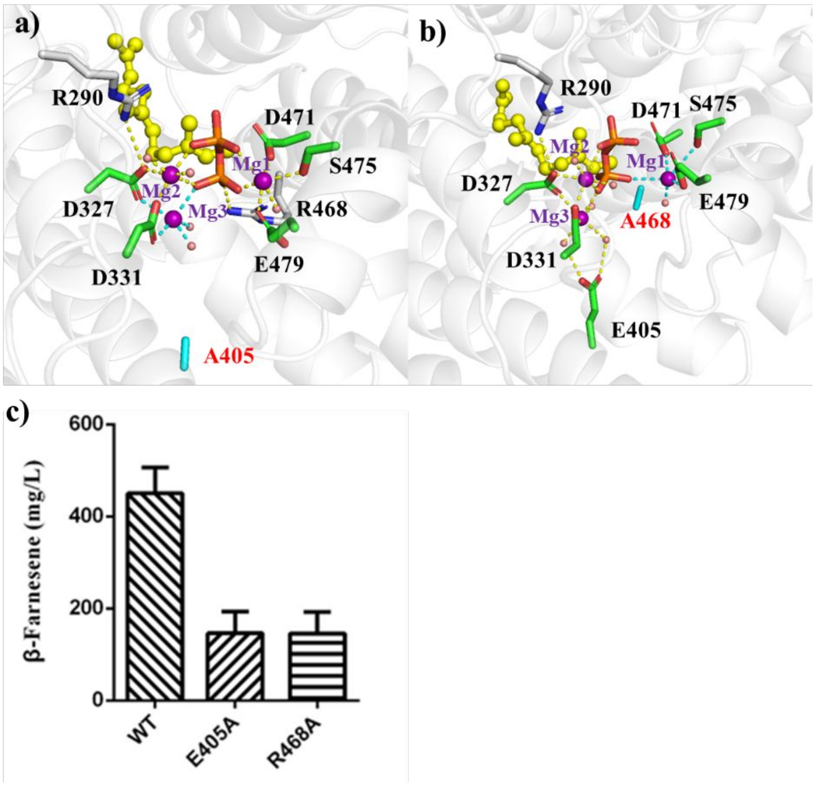


**Figure S2.** Binding environment of FPP in a) AaFS-E405A and b) AaFS-R468A. c) *β*-Farnesene yields of AaFS variants. Values represent the means of three biological replicates. Error bars are the standard deviations from these replicates.

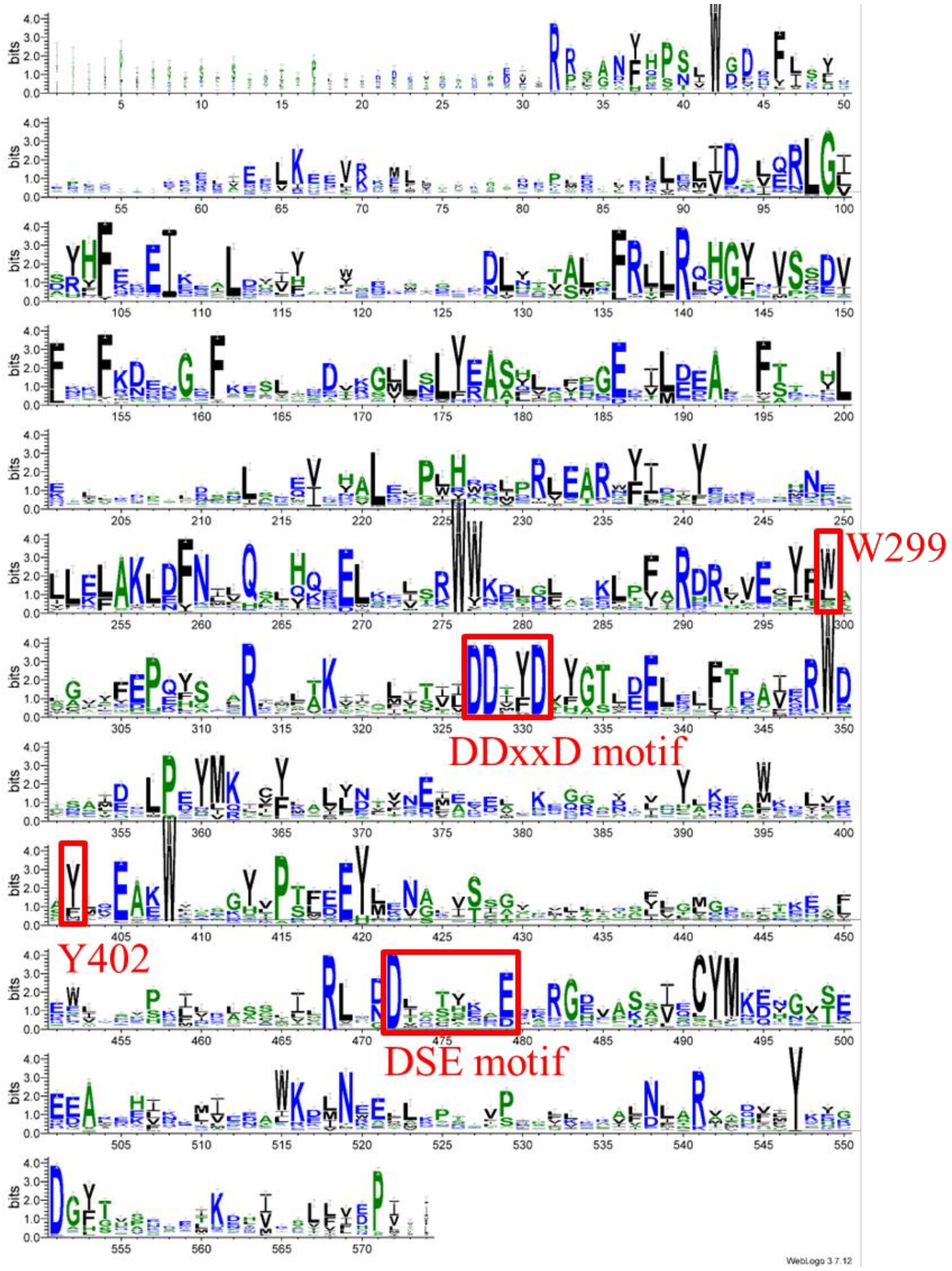


**Figure S3.** Weblogo plot based on multiple sequence alignment of 433 homologous sequences of AaFS from Uniplot database. The conserved DDxxD & DSE motifs and the key residues responsible for function modulation are highlighted in red boxes.

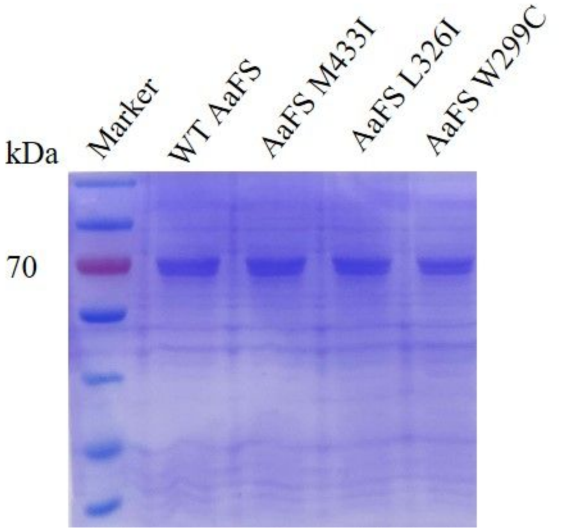


**Figure S4.** Electropherogram of the SDS-PAGE for AaFS and its variants.

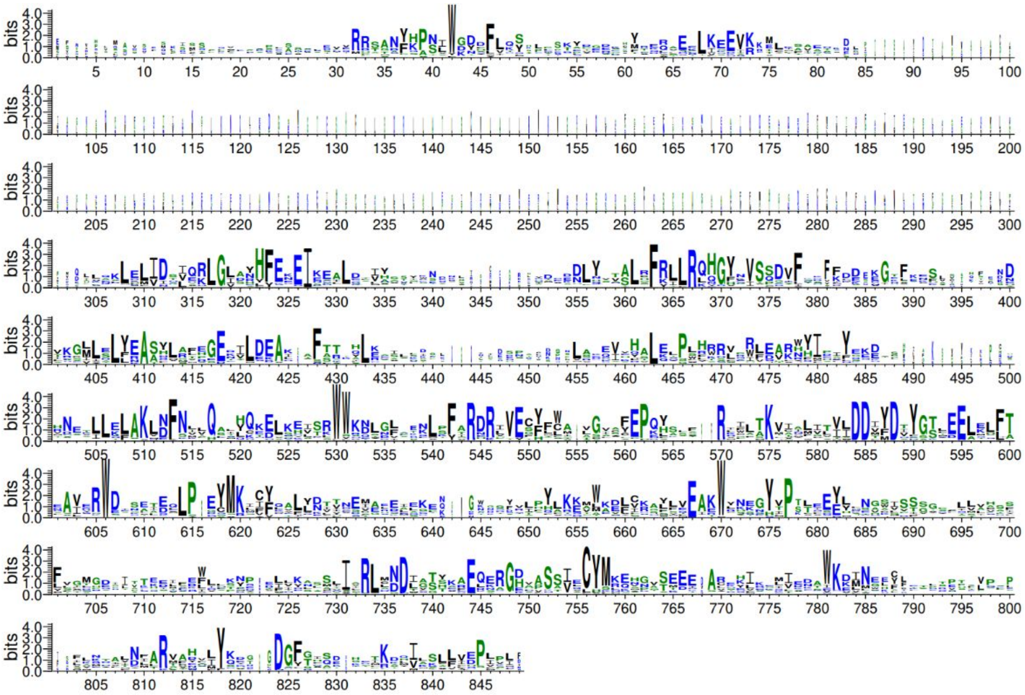

**Figure S5.** The propensity in amino acid residues in the sequences of AaFS and its 37 orthologues.

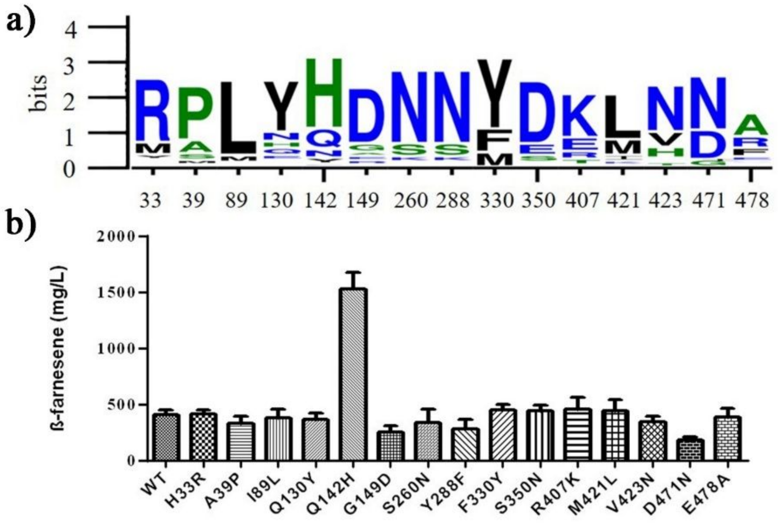


**Figure S6.** Site-directed mutagenesis of AaFS. a) Multiple sequence alignment between AaFS and its 37 orthologues. Weblogo plot revealed amino acid bias at the positions 33, 39, 89, 130, 142, 149, 260, 288, 330, 350, 407, 421, 423, 471 and 478. b) *β*-farnesene yields of AaFS variants. Values represent the means of three biological replicates. Error bars are the standard deviations from these replicates.

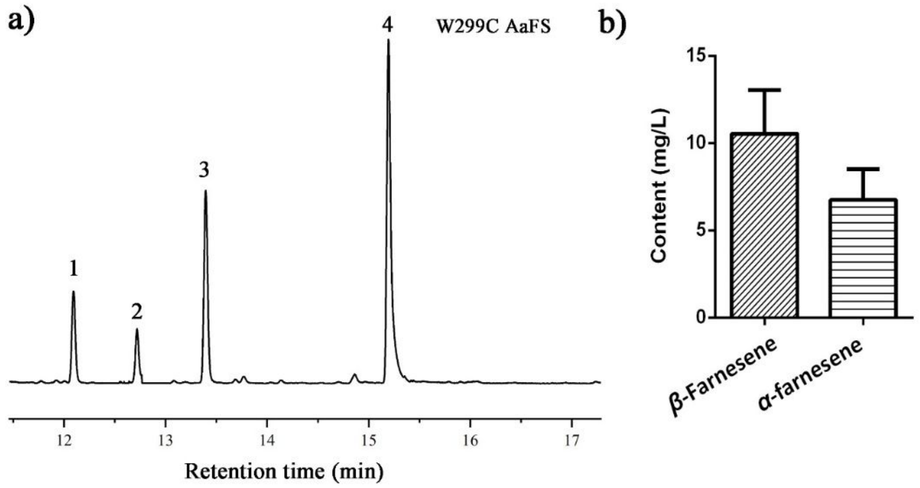


**Figure S7.** Product identification and farnesene production of AaFS W299C variant. a) Produced sesquiterpenes were identified as (1) $\beta$-farnesene; (2) $\alpha$-farnesene; (3) nerolidol; (4) farnesol. b) Farnesene production of AaFS W299C variant. Values represent the means of three biological replicates. Error bars are the standard deviations from these replicates.

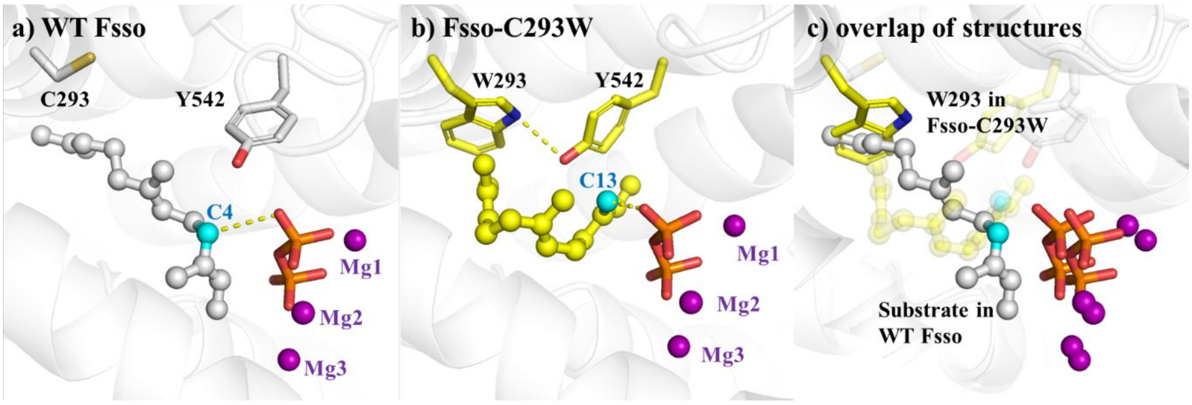


**Figure S8.** Conformational difference of the farnesyl cation substrate between Fsso-WT and Fsso-C293W. a) WT Fsso, b) Fsso-C293W, c) superimposition of WT and C293W variant of Fsso.

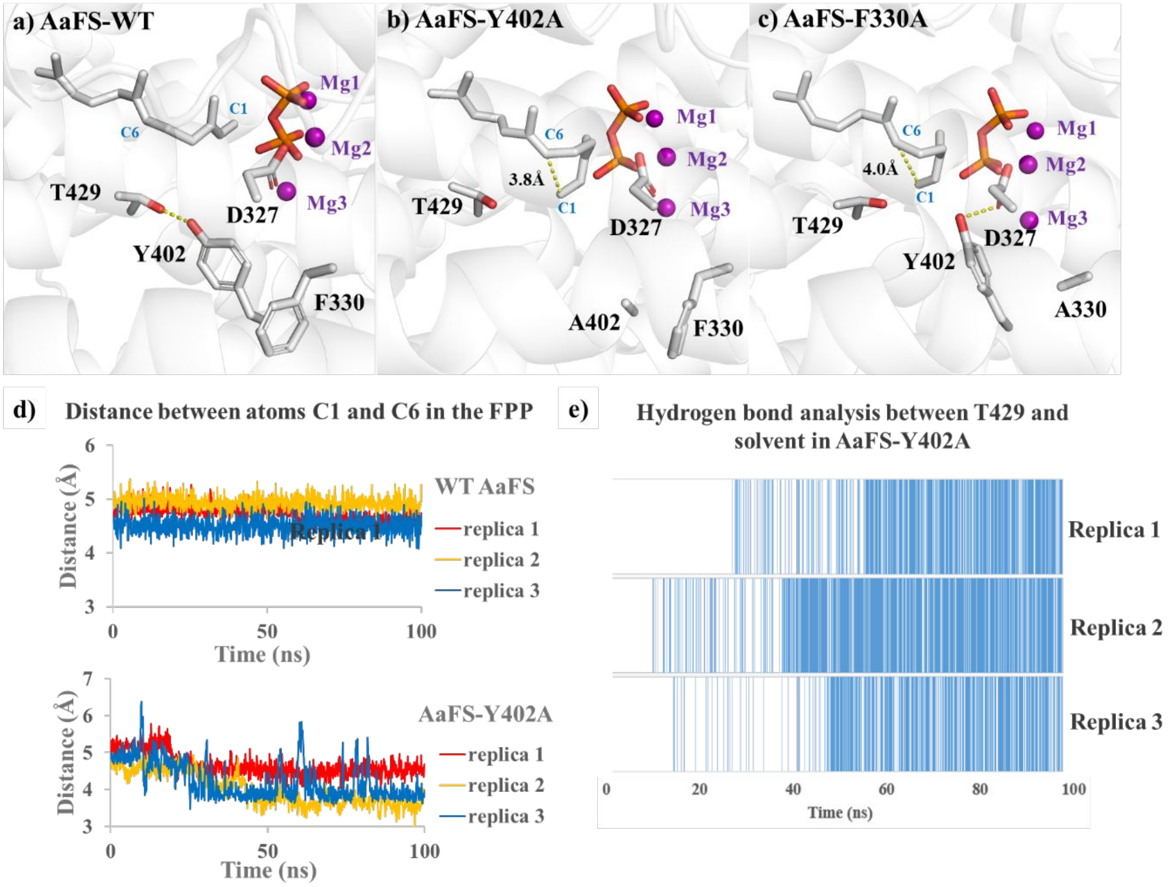


**Figure S9.** The substrate binding poses in AaFS WT and variants. a) AaFS-WT b) AaFS-Y402A and c) AaFS-F330A. d) The distance between C1 atom and C6 atom in AaFS-WT and AaFS-Y402A during MD simulations. e) The count of hydrogen bonds formed between T429 and the water in the MD simulation trajectory of AaFS-Y402A. Blue lines indicate the presence of hydrogen bonds.

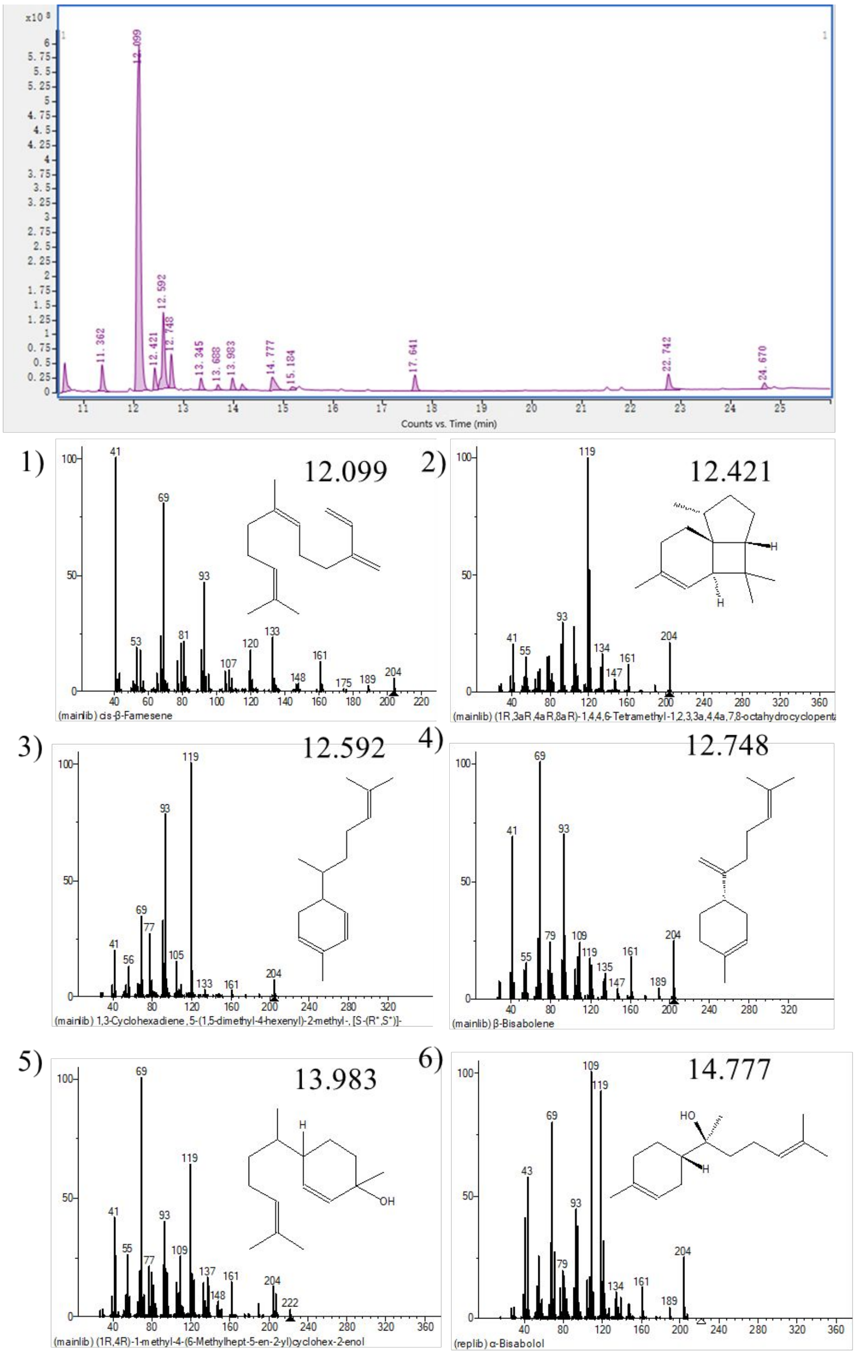


**Figure S10.** AaFS-F330A MS spectra corresponding to the sesquiterpene peaks. Produced sesquiterpenes were identified as (1) *β*-farnesene; (2) Isoitalicene; (3) Zingiberene; (4) *β*-bisabolene; (5) Zingiberenol; (6) *α*-bisabolol.
